# Influence of Water Source Quality on Concrete Performance: A Mechanism-Based Systematic Review and Engineering Evaluation Framework

**DOI:** 10.3390/ma19143077

**Published:** 2026-07-17

**Authors:** Kevin Paolo V. Robles, Jean Naethan Cordova, Maria Christyn Kinazo, Seong-Hoon Kee

**Affiliations:** 1 School of Civil, Environmental and Geological Engineering, Mapua University, Manila 1102, Philippines; jnjcordova@mymail.mapua.edu.ph (J.N.C.); mcbkinazo@mymail.mapua.edu.ph (M.C.K.); 2 Department of ICT Integrated Safe Ocean Smart Cities Engineering, Dong-A University, Busan 49315, Republic of Korea

**Keywords:** alternative water sources, water quality, concrete durability, cement hydration, wastewater reuse, sustainable concrete production

## Abstract

The use of alternative water sources in concrete production has gained increasing attention due to growing concerns over water scarcity and sustainability; however, existing studies predominantly evaluate water suitability based on compressive strength, often overlooking durability performance and underlying material behavior. This study presents a comprehensive systematic review of the influence of water source quality on concrete performance, integrating findings across fresh properties, mechanical behavior, durability indicators, and microstructural characteristics. The results indicate that concrete performance is primarily governed by the chemical composition, impurity concentration, and treatment level of the mixing water rather than its classification as potable or non-potable. While many studies report comparable compressive strength when water quality parameters are controlled, significant variations are observed in workability, durability, and microstructural development, highlighting the limitations of strength-based evaluation. To address these limitations, a mechanism-based interpretation framework is proposed, establishing a multi-scale relationship linking water chemistry, hydration reactions, microstructural development, transport properties, and durability performance, and providing a unified explanation for variability across studies. Based on this framework, water sources are further classified into low, moderate, and high impurity regimes, offering a structured basis for interpreting performance trends. In addition, a multi-dimensional engineering evaluation framework is developed, integrating fresh properties, mechanical performance, durability indicators, and microstructural characteristics to support practical decision-making in concrete production. Overall, this study advances current knowledge by transitioning from a descriptive review to a mechanism-based and framework-driven synthesis, bridging the gap between experimental findings and practical engineering application in sustainable concrete production.

## 1. Introduction

Concrete remains the most widely used construction material worldwide [1,2], and water is a critical component in its production [3]. Beyond enabling mixing, water initiates cement hydration, controls workability, and influences the development of strength and durability [4]. Because hydration depends on chemical reactions between cement compounds and water, variations in water chemistry—such as pH, dissolved solids, chlorides, and sulfates—can affect reaction rates and the formation of the internal concrete structure [5,6]. Research consistently shows that water quality directly influences setting, hardening, and strength development, highlighting its role as a key factor in concrete performance [3,7].

**Figure 1:**
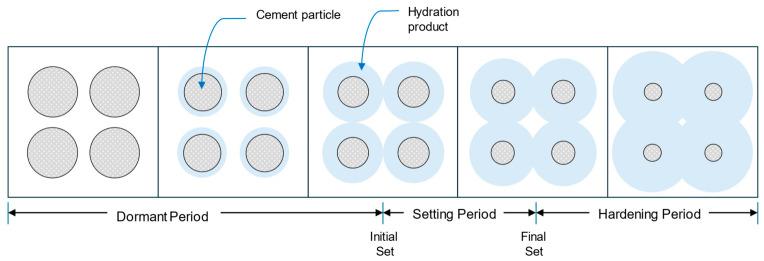
Author-illustrated schematic diagram of the cement hydration process and its stages, showing the interaction between cement particles and water leading to the formation of key hydration products such as calcium silicate hydrate (C–S–H) and calcium hydroxide.

As illustrated in Figure 1, the hydration process involves a series of complex chemical reactions between cement particles and water, leading to the formation of key compounds such as calcium silicate hydrate (C–S–H) and calcium hydroxide [8]. These hydration products gradually fill the spaces between particles, forming a dense microstructure that gives concrete its strength [9]. The diagram also emphasizes the sequential stages of hydration—from initial mixing and dormant period to setting and hardening—demonstrating how water governs each phase of the process. Importantly, the diagram highlights that any change in water composition can alter these reactions. For instance, impurities or high concentrations of certain ions may accelerate or retard hydration, disrupt the formation of hydration products, or lead to weaker and more porous microstructures [10]. This directly impacts not only early-age properties such as workability and setting time but also long-term performance, including compressive strength and durability.

The hydration process is governed by reactions between cement clinker phases and water, leading to the formation of calcium silicate hydrate (C–S–H) and calcium hydroxide (CH), which are primarily responsible for strength development and microstructural refinement [11,12,13]. Representative hydration reactions for tricalcium silicate (C_3_S) and dicalcium silicate (C_2_S) are presented in Equations (1) and (2), respectively.

(1) $$2C_{3}S\left(s\right)+6H_{2}O\left(l\right)\to C_{3}S_{2}H_{3}\left(s\right)+3Ca{\left(OH\right)}_{2}(s)$$

(2) $$2C_{2}S\left(s\right)+4H_{2}O\left(l\right)\to C_{3}S_{2}H_{3}\left(s\right)+Ca{\left(OH\right)}_{2}(s)$$

In addition to these primary hydration reactions, dissolved ions present in alternative water sources may participate in secondary physicochemical processes that influence durability performance [14,15]. Sulfate ions may contribute to ettringite formation, as shown in Equation (3), while chloride ions may react with hydrated aluminate phases to form Friedel’s salt, as illustrated in Equation (4). These representative reactions demonstrate that variations in water chemistry can influence hydration kinetics, hydration product formation, microstructural development, and long-term durability performance [16,17].

(3) $$C_{3}A\left(s\right)+3{CaSO}_{4}\cdot 2H_{2}O\left(s\right)+26H_{2}O(l)\to C_{6}{AŜ}_{3}H_{32}\left(s\right)$$

(4) $$C_{3}A\left(s\right)+{CaCl}_{2}\left(aq\right)+10H_{2}O\left(l\right)\to C_{4}AClH_{10}\left(s\right)$$

Therefore, understanding the relationship between water quality and cement hydration is essential for ensuring optimal concrete performance. It underscores the need for careful selection and control of mixing water in construction practices, as even minor variations can have significant effects on the structural integrity and longevity of concrete.

Although potable water is traditionally recommended to ensure predictable behavior, increasing pressure on freshwater resources has encouraged the use of alternative sources such as treated wastewater, recycled construction water, groundwater, and surface water [18,19]. Reviews of the use of reclaimed wastewater indicate that water containing controlled levels of dissolved contaminants can be incorporated without severe strength reduction when chemical limits are maintained [20]. However, differences in ionic composition may still influence hydration processes and lead to measurable variations in compressive strength. This shift toward alternative water sources reflects a broader need for sustainable resource management in construction practices [21,22].

Experimental studies further show that water impurities affect mineralogical composition and mechanical performance. For example, previous studies have reported that impurities present in mixing water contribute to changes in internal structure and strength development [3]. Similarly, investigations involving groundwater and saline-influenced sources demonstrate that chemical composition interacts with mixture proportions and curing conditions, producing variability in compressive strength outcomes [23,24].

Microstructural research helps explain these differences. Changes in pore distribution, hydration morphology, and transport properties have been associated with variations in water chemistry, which influence durability-related behavior [25,26]. In addition to hardened properties, water quality also affects fresh concrete behavior, including workability and setting characteristics, by modifying reaction kinetics and cement particle interactions [27]. These effects reflect both chemical parameters such as pH, salinity, and dissolved ions, and physical parameters including turbidity and suspended solids, which together shape fresh, hardened, and durability-related performance [28].

Despite extensive investigation into the use of alternative water sources in concrete production, existing studies remain fragmented and predominantly strength-oriented, with compressive strength widely adopted as the primary criterion for assessing water suitability [29,30,31,32]. While practical and widely accepted, such an approach provides only a partial representation of concrete performance because it often overlooks the physicochemical mechanisms governing hydration, microstructural development, durability, and long-term behavior [33,34]. Furthermore, variations in water characterization methods, mixture proportions, curing regimes, and testing procedures have contributed to inconsistencies in reported findings, limiting direct comparison across studies. Consequently, there remains no consolidated synthesis that systematically relates specific physical and chemical water quality parameters to fresh properties, mechanical behavior, durability performance, and microstructural characteristics within a unified analytical framework. A comprehensive review integrating these aspects is therefore needed to clarify areas of agreement, explain sources of variability, and identify methodological limitations in the existing literature.

In response to these limitations, this study advances a mechanism-based and framework-driven perspective that extends beyond conventional descriptive reviews. A multi-scale conceptual framework is proposed, linking water chemistry, hydration reactions, microstructural development, transport properties, and durability performance, thereby establishing a coherent cause-and-effect relationship across different performance domains. This integrated approach provides a scientifically grounded basis for interpreting experimental variability and evaluating alternative water sources in concrete production. In addition, it facilitates a more comprehensive understanding of how water quality influences concrete performance beyond traditional strength-based assessments.

Accordingly, this study aims to systematically evaluate the influence of water source quality on concrete performance by integrating findings related to fresh properties, mechanical behavior, durability indicators, and microstructural characteristics. Specifically, the review identifies the key physical and chemical parameters used to characterize water quality, examines their effects on hydration and concrete performance, assesses the suitability of testing methods employed in existing studies, and compares reported outcomes across different alternative water sources. The findings are expected to provide a more comprehensive basis for evaluating the use of non-potable water in concrete production, supporting improved material selection, standard compliance, and sustainable water management practices within the construction industry.

## 2. Review Methodology

### 2.1. Review Design

**Figure 2:**
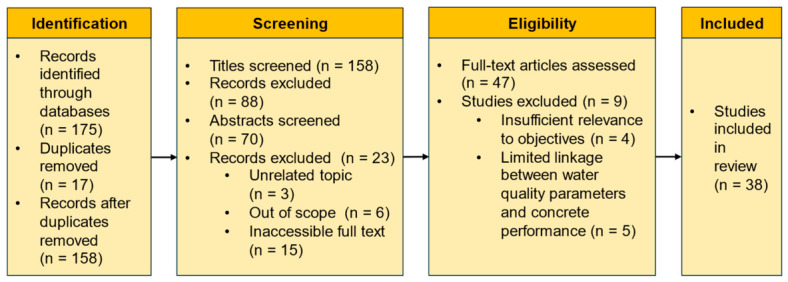
PRISMA flow diagram illustrating the identification, screening, eligibility assessment, and final inclusion of studies in the systematic review. Records were progressively filtered through duplicate removal, title and abstract screening, and full-text assessment based on the predefined inclusion, exclusion, and relevance assessment criteria.

This study was conducted using a Systematic Literature Review (SLR) to ensure a structured and transparent synthesis of existing research on the use of alternative water sources in concrete production. The research question and eligibility criteria were established prior to the literature search to ensure a structured and consistent selection process. The selection process followed the PRISMA 2020 framework, which organizes the review into four stages: identification, screening, eligibility assessment, and final inclusion of studies. Each stage was applied systematically to document how records progressed from the initial search results to the final set of included papers. A PRISMA flow diagram was prepared to clearly present the number of studies identified, screened, excluded, and included at each stage, as shown in Figure 2, ensuring transparency and methodological clarity throughout the review process. This approach enhances the reproducibility and reliability of the review by minimizing selection bias and ensuring a systematic evaluation of the literature. The completed PRISMA 2020 Checklist used to guide the reporting of this systematic review is provided in the Supplementary Materials.

### 2.2. Search Strategies and Study Selection

To identify relevant research on the influence of water quality and mixing water characteristics on concrete performance, a comprehensive search was conducted across five major electronic databases: Web of Science, IEEE Xplore, Scopus, ScienceDirect, and PubMed. An advanced search strategy was implemented using structured Boolean search strings composed of predefined keywords combined with logical operators (AND, OR) to ensure both comprehensive and focused retrieval of studies. The primary keywords included mixing water, water quality, water source quality, wastewater, concrete, cement, durability, mechanical properties, strength, and workability. The search strings were constructed according to each database’s indexing, syntax, and filtering rules, with minor modifications to optimize search performance across platforms. The detailed database-specific search strategies and the corresponding number of records initially retrieved are presented in Table 1.

The search was limited to publications from 2016 to 2026 to ensure inclusion of recent, relevant studies reflecting current practices, standards, and modern research approaches in concrete science. Where available, subject-area filters such as Engineering and Materials Science were applied to refine the search to studies directly related to civil engineering and construction materials. In IEEE Xplore, additional filters, including the availability of full-text PDF documents, were used to ensure accessibility of selected articles. The retrieved records formed the initial dataset, which was subsequently subjected to duplicate removal, screening, and eligibility assessment to ensure the inclusion of relevant and high-quality studies.

### 2.3. Data Extraction and Eligibility Assessment

Following the systematic search process, the identified records underwent a structured screening procedure, as illustrated in the PRISMA diagram (see Figure 2). This process consisted of identification, screening, eligibility assessment, and final inclusion to determine which studies were suitable for inclusion in the review.

From the 175 records obtained during the initial search, 17 duplicate entries were identified and removed. The remaining 158 studies proceeded to title screening, during which 88 records were excluded because they did not align with the main research focus. The remaining 70 articles were subjected to abstract screening. At this stage, studies focusing on replacing cement or aggregates with alternative materials were excluded to ensure methodological consistency throughout the review. Studies involving seawater were also excluded because chloride-induced corrosion mechanisms fall outside the scope of this review. Additional exclusions at the abstract level included unrelated topics (n = 3), out-of-scope studies (n = 9), and inaccessible full-text articles (n = 15). Following this process, 47 full-text articles were retained for eligibility assessment.

The 47 full-text articles were further evaluated using a structured relevance scoring system, summarized in Table 2. This five-point scale assessed each study based on its alignment with the review objectives and the clarity of the relationship between measurable water quality parameters and concrete performance outcomes. Studies assigned a score of 1 or 2 were excluded because they were deemed insufficiently relevant or lacked a strong analytical linkage. Only studies with scores ranging from 3 to 5 were included in the final synthesis. Following full-text eligibility assessment and scoring, 38 studies met the inclusion criteria and were included in the systematic review. This relevance-based filtering ensured that only studies with sufficient methodological rigor and direct relevance to the research objectives were considered in the final analysis. It should be noted that the total number of cited references exceeds the number of studies included in the PRISMA-based review, as additional sources were incorporated to support the introduction, research gap discussion, and recommended future directions.

It should be noted that the relevance scoring system was developed as a structured screening tool to evaluate the alignment of each study with the objectives of the present review and was not intended to serve as a formal risk-of-bias or quality assessment instrument. Due to the substantial heterogeneity in experimental methodologies, water quality characterization procedures, mixture designs, curing conditions, testing ages, and reported performance indicators among the reviewed studies, the applicability of standardized risk-of-bias assessment frameworks was considered limited. Consequently, study selection emphasized relevance, methodological consistency, and direct applicability to the review objectives rather than formal evidence grading.

Formal reporting bias assessment was also not conducted. The included studies primarily consisted of independent experimental investigations with diverse objectives, testing protocols, and outcome measures, which limited the applicability of conventional publication-bias assessment methods typically employed in quantitative evidence synthesis and meta-analyses. Similarly, a formal certainty-of-evidence assessment was not performed because the review aimed to qualitatively synthesize mechanistic relationships and performance trends rather than estimate pooled effect sizes or intervention outcomes. Given the diversity of experimental conditions and reported metrics, the use of standardized evidence-certainty frameworks was not considered appropriate.

Protocol registration was not undertaken because this review was designed as an engineering-focused systematic review intended to identify, synthesize, and interpret existing evidence regarding the influence of water source quality on concrete performance. The review followed predefined research objectives, eligibility criteria, search strategies, and study selection procedures established prior to data collection. Furthermore, the review process adhered to the PRISMA 2020 framework to promote transparency, reproducibility, and methodological rigor throughout the identification, screening, eligibility assessment, and inclusion stages.

Due to substantial differences in experimental methodologies, water quality characterization procedures, mixture designs, curing conditions, testing ages, and reported performance metrics, direct statistical comparison and quantitative normalization across all reviewed studies were not considered appropriate. Consequently, the present work adopts a qualitative synthesis approach focused on identifying recurring mechanistic relationships, performance trends, and knowledge gaps rather than performing a formal meta-analysis. Future reviews may benefit from the incorporation of inter-reviewer agreement measures, formal quality assessment protocols, protocol registration, and quantitative evidence synthesis techniques as the body of literature becomes more standardized.

### 2.4. Data Analysis

The selected studies were examined using a structured comparative framework to organize and synthesize reported findings on water source quality and concrete performance. Only studies utilizing Ordinary Portland Cement and natural aggregates were included, consistent with the defined scope of the review. Seawater and corrosion-dominated marine exposure mechanisms were excluded.

The synthesis of findings was conducted using a thematic analysis approach, wherein reported results were grouped into recurring performance domains to enable systematic comparison across studies.

Water quality parameters reported in the literature were first categorized into physical and chemical characteristics to enable consistent comparison across studies. Physical parameters included turbidity, suspended solids, and electrical conductivity, while chemical parameters included pH, total dissolved solids, chlorides, sulfates, alkalinity, and hardness.

Reported outcomes were then organized into three primary performance domains: fresh properties, mechanical properties, and durability-related indicators. Fresh properties included slump and setting time. Mechanical properties included compressive, tensile, and flexural strength, with emphasis on 7-day and 28-day compressive strength. Durability-related indicators included water absorption, permeability, electrical resistivity, and chloride penetration where reported.

Microstructural analyses, such as scanning electron microscopy, X-ray diffraction, and pore structure evaluation, were included where available to explain observed variations in mechanical and durability performance. However, these were treated as supporting evidence rather than as a standalone analytical category.

Methodological differences such as curing conditions, impurity concentrations, water replacement levels, and testing procedures were noted to ensure that comparisons were made within consistent experimental contexts. This structured analytical framework enabled a systematic comparison of findings across studies and supported the identification of consistent trends, variations, and methodological limitations within the literature.

## 3. Literature Characteristics and Testing Approaches

### 3.1. Research Trends and Keyword Analysis

**Figure 3:**
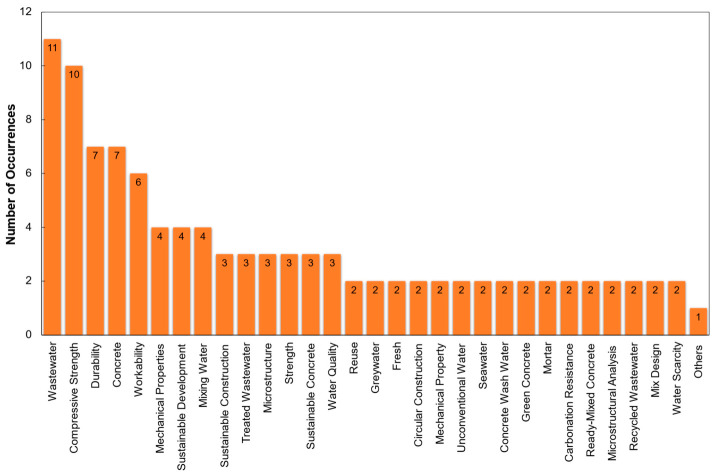
Frequency of Author-Provided Keywords in the Selected Studies.

A keyword frequency analysis was conducted to identify the dominant research themes in the reviewed literature. Among the 38 studies analyzed, the most frequently occurring keywords are “wastewater” (11 occurrences) and “compressive strength” (10 occurrences), followed by “durability” (7), “concrete” (7), and “workability” (6), as shown in Figure 3.

The frequent occurrence of the keyword ‘wastewater’ indicates that wastewater reuse represents a major research theme within the reviewed literature. This observation reflects the emphasis of the selected studies rather than the scope of the present review, which encompasses a broader range of alternative water sources including greywater, industrial wastewater, concrete plant wastewater, and natural water sources. This suggests that current research is strongly oriented toward wastewater reuse as a sustainable alternative in concrete production. In parallel, the prominence of the term “compressive strength” highlights that this is the most consistently assessed performance parameter. This reflects a research emphasis on mechanical strength as the primary indicator of material performance.

**Figure 4:**
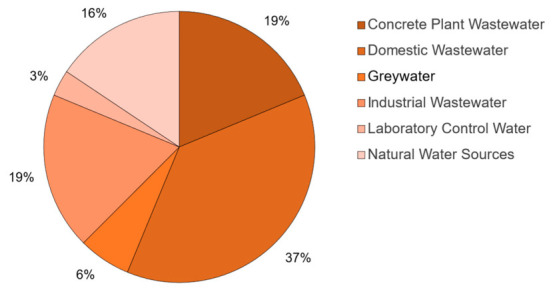
Distribution of Alternative Water Source Categories Used for Concrete Mixing in Reviewed Studies.

In addition, the recurring keywords “durability,” “concrete,” and “workability” provide further insight into the broader research focus. The presence of “durability” indicates that several studies extend their assessment beyond immediate strength performance to consider long-term behavior. However, its comparatively lower frequency suggests that durability-related assessments remain less consistently investigated compared to mechanical properties. Similarly, the keyword “workability” reflects research attention on the fresh-state properties of concrete when alternative water sources are used. Its lower occurrence further indicates that fresh-state properties are often treated as secondary considerations relative to strength performance. Figure 4 presents the distribution of alternative water source usage in the reviewed studies. Among the various categories, domestic wastewater is the most commonly utilized, representing approximately 37% of the total studies.

This indicates that municipal wastewater is the most extensively studied alternative water source in concrete research. Industrial wastewater (19%) and concrete plant wastewater (19%) also constitute significant portions of the literature. Their substantial representation reflects research efforts to repurpose water generated from industrial and construction processes rather than treating it solely as waste. This pattern suggests continued interest in evaluating these by-product water sources for practical application in concrete production.

Natural water sources account for about 16% of the studies, reflecting moderate research interest in non-potable but naturally available water. Although these sources are considered in comparative evaluations, they are examined less frequently than wastewater-based alternatives. In contrast, greywater (6%) and laboratory control water (3%) receive comparatively limited attention, highlighting potential gaps in the current research landscape.

### 3.2. Water Quality Parameters

To better understand the characteristics of water used in concrete production, relevant water quality parameters reported in previous studies were compiled and compared. The parameters presented in Table 3 were extracted from various research works investigating the use of different water sources such as tap water, treated wastewater, recycled water, greywater, industrial effluents, and natural water sources in concrete production.

The collected parameters include both physical and chemical indicators of water quality, such as total dissolved solids (TDS), total suspended solids (TSS), alkalinity, pH, sulfate concentration, chloride concentration, temperature, turbidity, electrical conductivity, chemical oxygen demand (COD), biological oxygen demand (BOD), and hardness. These parameters are commonly used to evaluate the suitability of water for concrete mixing and curing, as they directly influence hydration processes and long-term performance. By compiling these parameters across multiple studies, the table provides a comparative overview of how water quality varies depending on the source and treatment level.

Based on the data collected from the reviewed studies, several common water quality parameters are consistently reported in the literature. Among the most frequently analyzed properties are pH, total dissolved solids (TDS), sulfate concentration, chloride concentration, and total suspended solids (TSS). These parameters are widely measured because they directly influence cement hydration, the durability of concrete, and potential chemical reactions within the cement matrix [35,36]. For instance, pH is a critical indicator of alkalinity, as extreme pH levels may affect the setting time and stability of hydration products [36]. Likewise, TDS and TSS reflect the presence of dissolved and suspended impurities that can alter the water–cement ratio or interfere with hydration reactions [37]. Sulfate and chloride concentrations are also frequently assessed because of their potential to cause sulfate attack or reinforcement corrosion in reinforced concrete structures [36,38].

Water quality in concrete production is therefore primarily defined by a combination of physical and chemical parameters that represent both the purity and chemical composition of the water source. Key characteristics such as pH, TDS, TSS, sulfate, and chloride concentrations serve as primary indicators for determining whether water can safely participate in cement hydration without causing adverse reactions or long-term durability issues [35,39]. These parameters are generally classified into two main groups: physical properties and chemical properties. Physical parameters, including turbidity, temperature, and suspended solids, describe the clarity and particulate content of the water. In contrast, chemical parameters such as pH, dissolved salts, sulfate, chloride, COD, and BOD indicate the presence of dissolved compounds that may influence cement chemistry or structural durability [36,38].

Across the reviewed studies, these parameters are typically evaluated through standard laboratory analyses and compared with limits specified in established guidelines such as ASTM and other international standards for mixing water in concrete [40,41]. These evaluations provide a basis for determining whether alternative water sources meet acceptable thresholds for concrete production and for predicting their potential effects on mechanical properties and long-term durability.

Although a core set of parameters is commonly reported, other indicators appear less frequently in the literature. These include turbidity, temperature, hardness, electrical conductivity, COD, and BOD. While these parameters provide additional insight into contamination levels and organic content, they are not consistently included in concrete-related water quality assessments. For example, COD and BOD are more commonly associated with wastewater treatment studies but are occasionally examined in research involving recycled or industrial wastewater used in concrete. This inconsistency suggests that researchers prioritize parameters with direct influence on cement chemistry over broader environmental indicators.

The compiled data reveal significant variability in water quality depending on the source and level of treatment. Clean water sources such as potable or distilled water generally exhibit lower concentrations of TDS, TSS, and other contaminants, whereas wastewater, industrial effluents, and recycled water often contain higher levels of dissolved salts, suspended solids, and organic matter. These variations emphasize the need for systematic evaluation of water quality parameters prior to their use in concrete production to ensure that performance and durability are not compromised. Furthermore, the observed variability across studies highlights the lack of standardized reporting practices, which limits direct comparison and underscores the need for more consistent characterization of water quality in future research.

**Table 3:** Water Quality Parameters Across Studies.

Water Source Category	Reference	Specific Water Source	TDS (mg/L)	TSS (mg/L)	Alkalinity (mg/L)	pH	Sulfate (mg/L)	Chloride (mg/L)	Temp (°C)	Turbidity (NTU)	Conductivity (µS/cm)	COD (mg/L)	BOD (mg/L)	Hardness (mg/L)
	[42]	Bore water	269	10	302	7.5	0	70.9	—	0.01	148	6	1	138
Potable/Control Water Sources	[43]	Tap water	530.4	—	115	8	104	125	25	—	764	—	—	196
	[44]	Tap water	100–450	4	—	7.9–8.5	—	—	—	<5	—	0	0	—
	[45]	Well Water	454	—	—	7.3	117	106	—	—	709	—	—	—
	[46]	Distilled water	98	—	—	7	45	0.5	—	0	—	0	0	—
	[46]	Tap water	145	—	—	7	52	0.1	—	1	—	0	0	—
	[47]	Tap water	806	0	—	7.6	115.3	134.7	—	—	—	0	0	—
	[48]	Tap water	806	0	—	7.6	115.3	134.7	—	—	1.26 dS/m	0	0	385
	[49]	Tap water	—	—	—	7.1	9	18.3	—	—	—	—	—	—
	[50]	Drinking water	—	—	Trace	8.2	40.8	150	—	—	—	—	—	—
	[51]	Domestic water	—	28	—	7.03	45.5	24.91	—	—	—	—	—	—
	[52]	Tap water	—	—	—	8.16	—	484.8	—	0.1	—	—	—	—
	[52]	Potable water	—	17.2	58	6.6	3	2.5	24.5	4.73	—	114	—	—
	[52]	Drinking water	—	90	—	7.8	—	—	—	—	139.9 µS/cm	—	—	—
	[53]	Potable water	—	121	—	7.2	50	94	25	2	193	412	0	3
	[54]	Potable water	—	—	—	—	42.4	21	—	—	—	—	—	—
Treated Wastewater and Effluents	[42]	Treated wastewater	1110	21.33	252	7.27	170.7	175	—	5.92	1191	72	43.5	190
	[42]	Polished filtered wastewater	1110	11	250	7.26	162	166	—	4.56	1196	52	32	166
	[55]	STP-1 effluent	360	80	112	7.9	—	74.98	26.5	7.6	—	—	—	—
	[55]	STP-2 effluent	190	90	168	7.9	—	64.98	26.4	7.6	—	—	—	—
	[44]	Recycled wastewater	1500	50	—	6–9	—	—	—	75	—	100	50	—
	[56]	Treatment wastewater	—	—	—	—	62	93	—	—	—	—	—	—
	[52]	Secondary treated wastewater	—	—	—	7.48	—	269.91	—	0.305	1.59 mS/cm	—	—	20
	[52]	Tertiary treated wastewater	—	—	—	7	—	257.92	—	0.586	1.59 mS/cm	—	—	14
	[52]	Treated wastewater B	—	574	—	8.2	—	—	—	—	927 µS/cm	—	—	—
	[52]	Treated wastewater A	—	1161	—	7.3	—	—	—	—	1757 µS/cm	—	—	—
	[53]	Treated effluent	373.33	—	—	6.65	8.3	100.64	—	—	—	231.11	72	27
	[53]	Polished effluent	356	—	—	7.78	10.3	99.99	—	—	—	360	51.2	17.33
	[53]	Treated wastewater	—	25	—	7.92	80	1230	17	10	3950	1870	150	114
Greywater	[57]	Raw greywater	980	436	—	7.5	222	243	—	—	—	900	536	—
	[57]	Treated greywater	803	2	—	7.9	137	208	—	—	—	6.97	2.98	—
	[48]	Greywater	1184	80	—	7.9	153.7	269.5	—	—	1.85 dS/m	310	196	610
Raw Domestic/Municipal Wastewater	[46]	Wastewater	1580	—	—	7.2	198	32	—	182	—	325	200	—
	[56]	Raw wastewater	—	—	—	—	48	90	—	—	—	—	—	—
	[49]	Raw wastewater	—	—	—	12.1	16.2	237.1	—	—	—	—	—	—
	[50]	Sewage water	—	270	598	10.2	132.4	210	—	—	—	—	—	—
	[53]	Raw sewage	545.83	—	—	6.48	7.7	109.84	—	—	—	373.33	449.09	352
	[53]	Primary wastewater	—	451	—	7.68	145	740	17–19	800	4120	2541	3215	1240
	[54]	Primary clarifier effluent	—	—	—	—	55.3	96.1	—	—	—	—	—	—
	[54]	Bar screen effluent	—	—	—	—	55.3	116.9	—	—	—	—	—	—
Industrial Wastewater	[43]	Wash water	2500	—	835	11.36	320	125	23.5	690	—	48	23.6	750
	[45]	Raw waste wash water	7097	123,400	—	12.6	<5.0	49.7	—	—	11,854	3216	714	—
	[45]	Filtered WWW	2420	—	—	12.7	14.7	77.7	—	—	10,190	48.7	13.4	—
	[45]	Filtered neutralized WWW	1493	—	—	7.2	—	—	—	—	1533	39.5	10.9	—
	[47]	Treated industrial wastewater	1638	30	—	8.5	288.2	340.4	—	—	—	350	99	—
	[48]	Coal mining wastewater	—	—	—	8.9	12.6	—	—	91	1656	—	—	—
	[58]	Oil & gas produced water	—	—	—	6.71	126.16	230,202.06	—	—	—	—	—	—
	[59]	Treated distillery wastewater	—	—	—	7.5	—	—	—	—	—	—	42,565	11,337
Natural Water Sources	[46]	River water	510	—	—	8	250	45	—	4	—	80	35	—
	[46]	Lake water	612	—	—	7.5	300	40	—	4.5	—	90	40	—
	[50]	Groundwater	—	—	Trace	6.6	75.2	175	—	—	—	—	—	—
	[52]	Deep well water	—	16.8	32	6.5	6	27.5	24.7	3.83	—	240	—	—
	[52]	Rainwater	—	17.2	30	6.4	15	8.75	24.4	10.42	—	150	—	—
	[52]	River water	—	36.4	62	6.75	7	3.75	24.8	4.48	—	116	—	—
	[60]	River water	—	—	—	8	—	—	25	—	—	—	—	—
	[56]	Sludge wastewater	—	—	—	—	89.33	129	—	—	—	—	—	—
Combined Water	[47]	Combined water	1222	15	—	8.1	201.7	237.6	—	—	—	175	49.5	—
	[48]	Combined water	995	40	—	7.8	134.5	202.1	—	—	1.56 dS/m	155	98	497.5
Recycled Water	[51]	Recycled water CH1	—	52,360	—	7.73	54.28	30.88	—	—	—	—	—	—
	[51]	Recycled water CH2	—	23,160	—	7.48	57.22	24.11	—	—	—	—	—	—
	[51]	Recycled water CH3	—	9446	—	7.68	47.23	27.84	—	—	—	—	—	—
	[51]	Recycled water CH4	—	6235	—	7.65	44.54	28.2	—	—	—	—	—	—
	[51]	Recycled water CH5	—	1854	—	7.88	50.92	22.92	—	—	—	—	—	—
	[51]	Recycled water CH6	—	200	—	7.6	44.3	28.15	—	—	—	—	—	—
Concrete Plant Wastewater	[61]	Concrete truck wash wastewater	—	—	—	12.86	149.6	16	—	—	—	—	—	—
	[62]	Ready-mixed concrete plant wastewater	121,210	—	—	12	34	78	—	—	—	391	—	—

### 3.3. Experimental Testing Approaches Reported in the Literature

**Figure 5:**
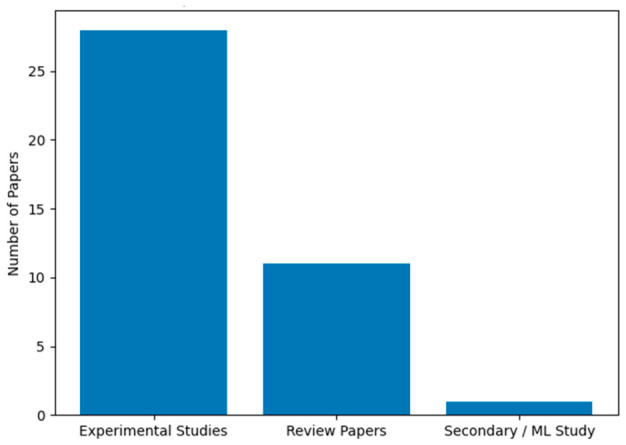
Composition of study types included in the reviewed literature.

The reviewed studies employ a variety of laboratory procedures to evaluate the influence of water quality on concrete performance. As shown in Figure 5, the dataset is dominated by experimental investigations, with a smaller number of review papers and secondary analyses. Given that experimental studies provide direct laboratory evidence, the present discussion focuses primarily on these investigations.

**Figure 6:**
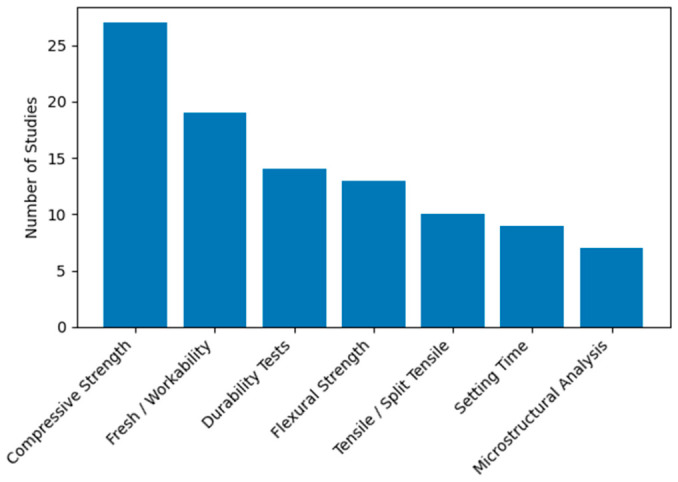
Frequency of testing methods reported across experimental studies.

The testing approaches reported in the literature can be broadly classified into fresh-state, mechanical, durability-related, and microstructural evaluations. Figure 6 summarizes the frequency of these testing methods across the reviewed experimental studies. Mechanical testing, particularly compressive strength assessment, is by far the most frequently employed approach, appearing in 27 studies. This predominance reflects the widespread use of compressive strength as the principal criterion for evaluating the suitability of alternative water sources in concrete production. Fresh-state evaluations, primarily through slump and workability tests, were reported in 19 studies, highlighting the importance of water chemistry in influencing mixture consistency and handling characteristics.

Additional mechanical properties, including flexural strength and tensile strength, were reported less frequently, appearing in 13 and 10 studies, respectively. While these tests provide valuable information regarding cracking behavior and structural performance, they remain secondary to compressive strength in most investigations. Durability-related assessments were reported in 14 studies and included water absorption, permeability, chloride penetration resistance, carbonation behavior, and corrosion-related indicators. Although these tests provide important insight into long-term performance, their lower frequency suggests that durability considerations remain less emphasized than strength-based evaluations.

Microstructural and mineralogical analyses, including SEM, XRD, and TGA, were reported in only seven studies. These techniques provide direct evidence of hydration processes, pore structure evolution, and microstructural development, offering mechanistic explanations for the performance trends observed in fresh and hardened concrete. However, their limited adoption indicates that relatively few studies investigate the underlying physicochemical mechanisms governing the effects of water quality.

Overall, the distribution of testing methods reveals a clear imbalance in current research priorities. Most studies focus on verifying the mechanical suitability of alternative water sources through compressive strength testing, whereas comparatively fewer investigations evaluate durability performance and microstructural behavior. This imbalance highlights the need for more comprehensive assessment frameworks that integrate strength, durability, and mechanistic analyses to better understand the long-term implications of using alternative water sources in concrete production.

## 4. Effects of Water Quality on Concrete Performance

### 4.1. Water Quality Effects on Concrete

To provide a clearer comparison of the findings from the reviewed studies, the key results related to concrete performance using different water sources are summarized in Table 4. The table presents the reported effects of various mixing water types on fresh properties (workability and setting time), as well as mechanical properties, including compressive, tensile, and flexural strength. By consolidating findings from multiple studies, the table enables a comparative evaluation of performance trends and highlights the variability in concrete behavior associated with different water sources and treatment conditions.

The summarized results reveal that the influence of water quality on concrete performance is not uniform and depends on factors such as impurity concentration, treatment level, and water replacement ratio. Across the reviewed studies, both improvements and reductions in performance have been reported, indicating that the suitability of alternative water sources cannot be generalized without considering their chemical and physical characteristics.

In general, treated or controlled water sources tend to produce concrete with performance comparable to that of potable water, while untreated or highly contaminated sources are more likely to result in reductions in workability, strength, and durability. However, variations in experimental conditions, including mixture proportions, curing regimes, and testing methods, contribute to differences in reported outcomes across studies.

To better interpret these findings, the effects of water quality are discussed in terms of key performance domains, including workability, setting time, mechanical strength, and durability-related behavior. This approach allows for a more structured comparison of results and provides a clearer understanding of how specific water quality parameters influence concrete performance.

#### 4.1.1. Workability

Workability is strongly influenced by the chemical composition, impurity concentration, and treatment level of the mixing water. Across the reviewed studies, alternative water sources generally produced either comparable or moderately reduced slump values relative to potable water, although improvements were reported in selected cases. Reductions in workability were commonly observed when treated wastewater, greywater, produced water, and concrete plant wastewater were used, with reported decreases ranging from approximately 13% to 50% depending on the water source and treatment level [40,43,45,57]. For example, Elsayed et al. reported a slump reduction of approximately 29% when tertiary treated wastewater replaced tap water [33], while Meena and Luhar observed decreases from 80 mm to 60 mm and 40 mm when tertiary and secondary treated wastewater were used [56].

Despite these reductions, many studies demonstrated that acceptable workability can still be achieved when water quality is adequately controlled. Treated domestic wastewater, polished filtered wastewater, recycled water, wash water, and various natural water sources generally produced slump values within acceptable ranges for concrete production [43,46,51]. In some cases, workability improvements were also reported, such as the higher slump values observed when concrete wash wastewater was used [50]. These findings suggest that workability is governed not simply by whether a water source is potable or non-potable, but by the type, concentration, and treatment of the impurities present.

The observed variations are primarily attributed to differences in dissolved solids, suspended particles, and organic compounds, which alter paste viscosity, particle lubrication, and the rheological behavior of fresh concrete [39,43]. Reduced workability may hinder proper compaction and increase the likelihood of defects such as honeycombing and entrapped air voids, ultimately affecting strength development and long-term durability performance [52].

#### 4.1.2. Setting Time

Setting time is highly sensitive to water chemistry, with both delayed and accelerated hydration reported across the reviewed studies. Delayed setting was commonly observed when alternative water sources contained organic matter or impurities, with increases of approximately 20–25 min reported for raw and treated greywater [33] and longer setting periods observed for recycled water, wash water, and treated wastewater [31,41,50]. In contrast, accelerated setting was reported when water sources contained salts or chemically active dissolved constituents, including produced water, sewage water, and wastewater containing chemical residues [36,53,54].

These contrasting trends indicate that the effect of alternative water on setting behavior depends primarily on contaminant type and concentration. Organic compounds may retard hydration by delaying hydration product formation, whereas certain dissolved salts and ions can accelerate hydration reactions [61]. Therefore, setting time should be evaluated based on the specific chemical composition of the mixing water rather than its broad classification as potable or non-potable.

#### 4.1.3. Compressive Strength

Compressive strength is the most frequently evaluated performance indicator in studies investigating the use of alternative water sources in concrete production. Overall, the reviewed studies indicate that compressive strength is governed more by impurity concentration, treatment level, and chemical composition than by whether the water source is classified as potable or non-potable. Treated or controlled water sources generally produced compressive strengths comparable to or higher than those obtained with potable water, with several studies reporting strength improvements ranging from approximately 5% to 79% [40,56,58,62]. In many cases, treated wastewater, recycled water, and properly managed industrial wastewater achieved strength levels similar to conventional concrete, particularly at later curing ages [43,45,59,60].

In contrast, untreated or highly contaminated water sources were more likely to result in strength reductions. Reported decreases ranged from approximately 10% to 37%, although some studies observed that later-age strengths approached those of control mixtures as hydration progressed [39,45,53,63]. The variability in reported performance reflects differences in water quality characteristics, replacement ratios, treatment processes, mixture proportions, and curing conditions, indicating that strength performance cannot be generalized solely on the basis of water source classification.

The observed strength reductions are commonly associated with increased porosity, higher water absorption, and the presence of aggressive ions such as sulfates. Sulfate ions may react with hydration products to form expansive compounds such as ettringite, generating internal stresses that promote microcracking and weaken the cement matrix. Similarly, increased pore connectivity reduces the ability of concrete to resist applied loads while facilitating the ingress of moisture and aggressive agents [66]. Consequently, the findings suggest that compressive strength is strongly influenced by the type, concentration, and treatment of impurities present in the mixing water, highlighting the importance of comprehensive water quality evaluation when assessing the suitability of alternative water sources for concrete production.

#### 4.1.4. Tensile and Flexural Strength

Tensile and flexural strength generally follow trends similar to those observed for compressive strength. Arooj et al. [65] reported tensile strength increases of approximately 20–25% when treated domestic wastewater was used. Elsayed et al. [33] also reported a slight increase of approximately 4.2% in tensile strength when tertiary treated wastewater was used. Reductions in tensile strength were also observed in some studies. Nasseralshariati et al. [67] reported reductions of approximately 19%, while Morgado et al. [53] reported decreases ranging from 5% to 37%. Jahandideh et al. [45] also reported reductions of up to 10%.

Flexural strength results show similar variations. it is reported improvements in flexural capacity ranging from 8.7% to 17.4% when coal mining wastewater was used [31]. Tsardaka et al. [42] also reported flexural strengths between 6.7 and 10.2 MPa when concrete wash wastewater was used. Decreases were reported by Meena and Luhar [56], who observed reductions between 7.7% and 13.1%. Jahandideh et al. [45] reported reductions of approximately 18–20% in beam bending strength when recycled wastewater was used in reinforced concrete. These variations indicate that flexural performance, like tensile strength, is sensitive to changes in microstructure and internal bonding within the concrete.

### 4.2. Mechanistic Interpretation of Water Quality Effects

To provide a deeper understanding of the observed trends across the reviewed studies, the influence of water quality on concrete performance is interpreted through a mechanistic cause–effect framework, linking water chemistry to material behavior across multiple scales. Rather than treating performance outcomes as isolated observations, this approach explains how variations in water composition propagate through hydration processes, microstructural development, and transport properties to ultimately influence durability performance.

At the initial stage, water chemistry directly governs cement hydration kinetics. The presence of dissolved ions such as chlorides, sulfates, and alkalis can significantly alter reaction rates and hydration pathways. Chloride ions may accelerate early hydration reactions, contributing to increased early-age strength, while excessive sulfate concentrations promote the formation of expansive products such as ettringite, which may destabilize the cement matrix over time [36,38]. Organic contaminants and suspended solids may adsorb onto cement particle surfaces, forming barriers that delay hydration and prolong setting time, as reflected in studies reporting delayed setting behavior when wastewater or greywater is used [30,36,52].

These changes in hydration behavior directly influence microstructural development, particularly the formation, distribution, and connectivity of pores within the cement matrix. Controlled levels of fine particles and dissolved solids may contribute to a filler effect, enhancing particle packing and refining pore structure. In contrast, high concentrations of impurities disrupt the formation of calcium silicate hydrate (C–S–H), leading to increased porosity and weaker interfacial bonding [25,26]. This explains why some studies report improved or comparable strength with treated water sources, while reductions are observed when untreated or highly contaminated water is used [50,54].

The resulting pore structure governs the transport properties of concrete, including permeability, water absorption, and ion diffusivity. A refined pore structure reduces connectivity and limits the ingress of aggressive agents, whereas increased porosity enhances fluid transport and accelerates deterioration mechanisms [25,26]. These transport characteristics form the critical link between microstructure and long-term performance.

Ultimately, these mechanisms determine durability performance, including resistance to chloride penetration, sulfate attack, and other degradation processes. Several studies reviewed in this work indicate that concrete mixtures may achieve acceptable compressive strength while still exhibiting reduced durability due to increased pore connectivity and transport properties [60].

**Figure 7:**
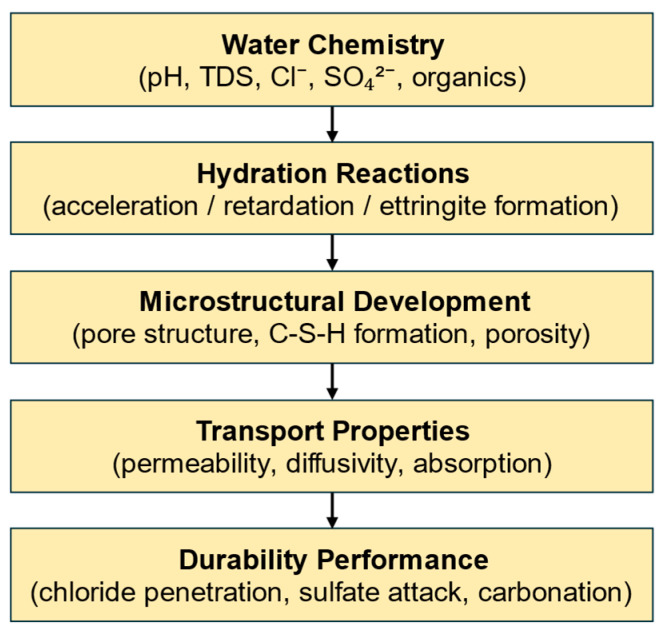
Multi-scale conceptual framework linking water chemistry to concrete performance.

Based on this interpretation, the influence of water quality on concrete performance can be systematically described through the casual chain shown in Figure 7. This framework provides a unified explanation for the variability observed across the literature and highlights the importance of evaluating water quality beyond strength-based indicators.

### 4.3. Classification of Water Quality Regimes and Performance Implications

To further interpret the variability in reported results across the reviewed studies, water quality can be classified into distinct regimes based on impurity concentration and composition. This classification is derived from the comparative trends observed in Table 5 and provides a structured framework for linking water quality to concrete performance outcomes.

In the low impurity regime, water sources contain minimal dissolved solids, low concentrations of aggressive ions, and negligible organic contaminants. Under these conditions, hydration reactions proceed without significant interference, resulting in the formation of a dense and well-developed microstructure. This leads to reduced permeability and improved durability performance, consistent with studies reporting comparable behavior between treated water and potable water [43,45,60].

The moderate impurity regime includes water sources with controlled but noticeable levels of dissolved salts and fine particles. In this range, hydration behavior becomes more variable depending on the specific composition of impurities. Some studies report enhanced or comparable mechanical performance due to filler effects or accelerated hydration, while others observe slight reductions in workability and durability. This variability reflects the competing effects of beneficial and detrimental interactions within the cement matrix [44,46,56].

In contrast, the high impurity regime is characterized by elevated concentrations of chlorides, sulfates, organics, and suspended solids. These conditions disrupt hydration processes, increase porosity, and weaken the cement matrix. As a result, transport properties are significantly enhanced, facilitating the ingress of aggressive agents and leading to reduced durability performance. This behavior is consistent with studies reporting strength reduction, delayed setting, and increased permeability when untreated or highly contaminated water sources are used [47,50,54].

This classification framework provides a systematic explanation for inconsistencies observed across experimental studies and supports a more consistent interpretation of water quality effects. It also reinforces the need to evaluate both impurity concentration and chemical composition when assessing the suitability of alternative water sources in concrete production.

## 5. Integrated Interpretation and Comparative Analysis

### 5.1. Comparative Study Findings

While the previous subsection discussed the mechanisms through which water quality parameters influence concrete behavior, this section compares the experimental findings reported across the reviewed studies to identify consistent performance trends. The reviewed literature presents a range of findings regarding the influence of alternative water sources on concrete performance. When studies are compared across compressive strength, fresh concrete behavior, durability indicators, and microstructural observations, several recurring patterns emerge. Many investigations report that treated or controlled non-potable water sources can produce concrete with mechanical performance comparable to mixtures prepared with potable water. At the same time, the results across the literature show considerable variation depending on the type of water source, treatment level, and mixture design. These differences indicate that the influence of water quality cannot be evaluated solely from compressive strength results and must also be examined in relation to fresh-state behavior, durability performance, and microstructural characteristics.

Across the reviewed studies, compressive strength remains the dominant parameter used to assess the suitability of alternative water sources. A consistent trend across the literature is that compressive strength is primarily governed by impurity type, concentration, and treatment level rather than whether the water source is potable or non-potable. Studies such as [32,44,45,47,57,59] demonstrate that treated wastewater and controlled water sources can achieve strength values comparable to control mixtures.

In contrast, untreated or highly contaminated water sources frequently lead to strength reductions. Studies such as [50,54] reported reductions of up to one-third when untreated wastewater was used. These differences are largely attributed to the presence of sulfates, chlorides, and organic matter, which interfere with hydration reactions and increase porosity.

In addition, mixture design and curing conditions play a significant role in moderating these effects. The use of supplementary cementitious materials and extended curing periods has been shown to reduce strength differences, indicating that water quality effects should not be evaluated in isolation.

Fresh concrete behavior, on the other hand, particularly workability, shows greater variability across the reviewed studies. Some studies report acceptable workability when alternative water sources are used. Workability is highly sensitive to the presence of suspended solids and dissolved impurities, making it one of the earliest indicators of water quality effects. While studies such as [43,50,59] report acceptable slump values when mixture proportions are adjusted, other studies consistently show reduced workability when untreated or highly contaminated water is used.

This variability is primarily caused by changes in paste viscosity and the availability of free water within the mixture. Dissolved solids and fine particles increase internal friction, reducing flowability. These findings indicate that workability depends on water quality.

Moreover, durability-related findings reveal a more complex pattern compared to compressive strength. Some studies report acceptable durability when strength is acceptable. A key observation across the literature is that acceptable compressive strength does not necessarily guarantee adequate durability performance. Studies such as [64,67] show that concrete can maintain strength while exhibiting reduced resistance to carbonation, permeability, or other durability-related properties.

This discrepancy is primarily linked to microstructural changes, particularly increased pore connectivity and transport properties. While some studies report acceptable durability when water is properly treated [40]. untreated or poorly controlled water sources generally result in higher porosity and reduced long-term performance.

These findings highlight that durability is more sensitive than compressive strength to variations in water quality and should be considered a critical parameter in evaluating suitability.

Microstructural observations provide critical explanations for the variations observed in mechanical and durability performance. Some studies show differences in pore structure. Across the reviewed studies, microstructural analysis consistently shows that water quality influences hydration products, pore structure, and matrix density. Treated water sources tend to produce denser and more uniform microstructures, while contaminated water leads to increased porosity and weaker bonding.

These findings confirm that the effects of water quality are not limited to surface-level performance but are fundamentally linked to changes in hydration mechanisms and internal concrete structure. Intrusion porosimeter showed that better-treated water suppressed unstable hydration phases and promoted a more refined cement matrix.

Other studies report similar relationships from different perspectives. Reference [54] linked reduced durability performance in primary-stage wastewater mixtures to increased porosity and weaker microstructural development. Ref. [44] suggested that certain dissolved solids in recycled tertiary wastewater may contribute to pore filling and improved strength development, particularly at later ages. In contrast, ref. [50] attributed the lower strength of sewage water concrete to increased porosity and entrapped air. Collectively, these findings show that water chemistry affects concrete performance not only through direct chemical interaction with cement but also through its influence on hydration products and pore development.

To synthesize the trends identified across the reviewed studies, Table 6 presents a matrix summarizing the typical performance outcomes associated with different categories of alternative water sources. Rather than listing individual experimental studies, the matrix consolidates the broader patterns observed in the literature by grouping water sources according to their typical strength outcomes, fresh concrete behavior, and durability observations. This approach allows the overall relationship between water source type and concrete performance to be interpreted more clearly.

Beyond the durability indicators directly reported in the reviewed studies, the observed changes in permeability, porosity, and transport properties have important implications for long-term deterioration mechanisms in reinforced concrete. Studies involving highly contaminated or untreated water sources consistently reported increased pore connectivity, reduced microstructural refinement, and higher transport characteristics, which may facilitate the ingress of aggressive agents and accelerate deterioration processes [39,43,52]. Elevated chloride concentrations commonly reported in wastewater-based sources may increase susceptibility to chloride ingress and subsequent reinforcement corrosion, particularly when combined with increased permeability and reduced resistance to ion transport [39,43,52]. Similarly, high sulfate concentrations may contribute to progressive durability degradation through disruption of hydration processes and deterioration of the cement matrix, resulting in reduced long-term performance [39,43,52]. These findings further suggest that acceptable compressive strength does not necessarily guarantee adequate long-term durability, as several studies reported satisfactory mechanical performance despite changes in transport properties and microstructural characteristics [33,35,54]. Consequently, the suitability of alternative water sources should be evaluated not only on the basis of strength performance but also with consideration of durability-related deterioration mechanisms, transport properties, and their potential implications for the long-term service life of reinforced concrete structures [33,35,39,43,52,54].

**Figure 8:**
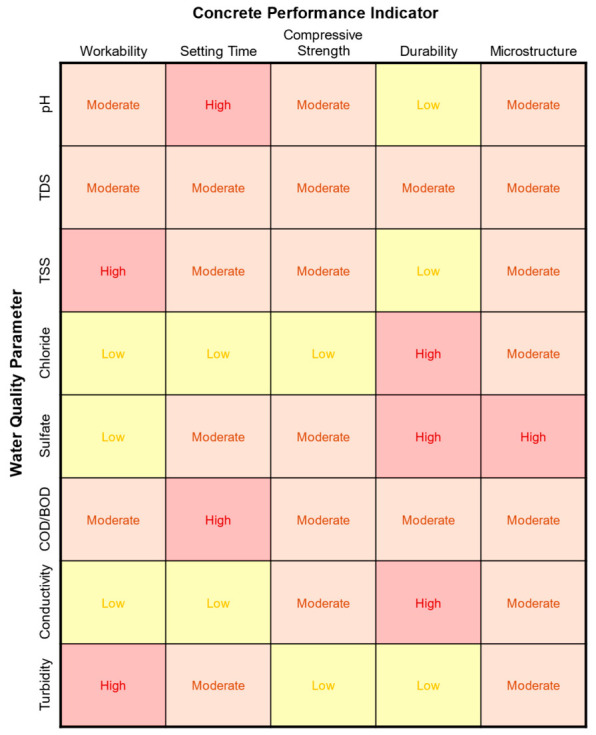
Qualitative mechanistic relationship heat map between key water quality parameters and concrete performance indicators. The classifications are based on recurring trends and mechanistic interpretations reported in the reviewed literature and are intended as a qualitative synthesis rather than a quantitative ranking.

The comparative analysis demonstrates that the influence of water quality cannot be evaluated using a single performance indicator. Instead, different water quality parameters affect concrete behavior through distinct mechanisms and with varying levels of influence across fresh, mechanical, durability, and microstructural properties. To synthesize these recurring relationships, Figure 8 presents a qualitative mechanistic heat map derived from the reviewed literature. The heat map highlights that chloride and sulfate concentrations exhibit the strongest influence on durability-related performance, whereas parameters such as turbidity and total suspended solids (TSS) primarily affect fresh concrete properties, particularly workability. In contrast, parameters such as pH, total dissolved solids (TDS), and organic content (COD/BOD) demonstrate broader influences across multiple performance domains through their effects on hydration kinetics and microstructural development.

The findings consistently show that properly treated or controlled water sources can be used in concrete production without significant loss of performance, while untreated or highly contaminated water introduces variability and potential risks. This synthesis highlights the need for standardized water quality characterization and comprehensive testing approaches that include not only compressive strength but also durability and microstructural evaluation to ensure reliable and sustainable use of alternative water sources in concrete production.

### 5.2. Quantitative Synthesis

**Figure 9:**
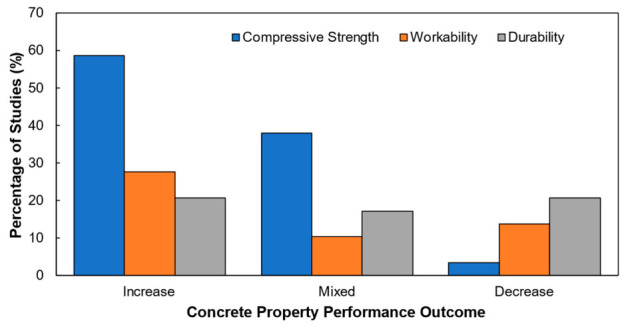
Summary of Reported Performance Outcomes Across Reviewed Studies.

To complement the qualitative comparison, a quantitative synthesis of the reviewed studies was conducted based on the filtered dataset, as shown in Figure 9. The results were categorized according to reported performance outcomes, including increases, comparable behavior, or reductions in key concrete properties. For compressive strength, approximately 58.6% of the studies reported improved performance when alternative water sources were used, while 37.9% showed mixed or comparable results, and only 3.4% reported clear reductions. These results indicate that compressive strength is generally not adversely affected when water quality is properly controlled.

In contrast, workability results show greater variability. Only 27.6% of studies reported improved workability, while 13.8% reported reductions and 10.3% showed mixed outcomes. A significant proportion (48.3%) did not report clear workability trends, suggesting inconsistent evaluation practices across studies.

Durability-related performance exhibited the highest variability. Approximately 20.7% of studies reported improved durability, while an equal proportion reported reductions. Around 17.2% showed mixed outcomes, and 41.4% of studies did not include durability evaluation. This indicates that durability remains insufficiently investigated compared with mechanical performance.

The quantitative synthesis confirms that compressive strength is the most consistently favorable parameter when alternative water sources are used, while workability and durability are more sensitive to variations in water quality. These findings reinforce the need for comprehensive evaluation beyond strength-based assessment when determining the suitability of non-potable water in concrete production.

## 6. Engineering Evaluation Framework for Water Quality in Concrete

The findings of this review demonstrate that the suitability of alternative water sources in concrete production cannot be reliably assessed using compressive strength alone. While many studies report comparable strength performance, significant variations are observed in workability, durability, and microstructural characteristics. These inconsistencies highlight the need for a more comprehensive and application-oriented evaluation approach.

To address this limitation, a multi-dimensional engineering evaluation framework is proposed, integrating performance indicators across fresh properties, mechanical behavior, durability, and microstructural characteristics. As summarized in Table 7, each evaluation domain captures a distinct aspect of concrete behavior and collectively provides a more holistic assessment of water suitability. Fresh properties, such as workability and setting time, serve as early indicators of changes in mixture behavior due to variations in water chemistry. Mechanical properties, including compressive, tensile, and flexural strength, reflect structural capacity but may not fully capture long-term degradation mechanisms. Durability indicators, such as permeability, water absorption, and resistance to chloride penetration, are critical for assessing service life, while microstructural characteristics provide mechanistic insight into the observed performance trends.

It should be noted that the proposed framework is intended as a mechanistic synthesis of recurring relationships reported across the reviewed studies rather than a statistically validated predictive model. The substantial variability in experimental methodologies, water quality characterization, mixture designs, and performance evaluation procedures among the reviewed studies currently limits the feasibility of a rigorous quantitative meta-analysis.

This multi-dimensional approach recognizes that satisfactory performance in one domain does not necessarily guarantee acceptable behavior in others. In particular, compressive strength may remain within acceptable limits even when durability performance is compromised due to increased porosity and transport properties. The framework therefore emphasizes the need to evaluate water quality based on combined performance outcomes rather than relying on a single indicator.

In addition to performance-based evaluation, a threshold-based classification of water quality parameters is proposed to support practical decision-making. The reviewed studies consistently identify total dissolved solids (TDS), chloride concentration, sulfate concentration, and pH as key indicators influencing hydration behavior and long-term durability. Based on these parameters and the classification framework presented in Section 4.2, water sources can be categorized into low, moderate, and high impurity regimes. The corresponding engineering recommendations are presented in Table 8, which provides a simplified decision-making guide for practical application.

The proposed threshold ranges were synthesized from reported values across the reviewed studies (Table 3) and aligned with commonly accepted limits in concrete practice. While variations exist depending on mixture design and exposure conditions, these ranges provide a practical engineering basis for preliminary water quality assessment.

As shown in Table 7, the suitability of alternative water sources is strongly dependent on impurity levels and corresponding impacts on hydration and microstructural development. Water sources within the low impurity regime can generally be used without modification, while moderate impurity conditions require controlled use and mix adjustments to maintain performance. In contrast, high impurity levels are associated with increased risks of durability degradation and therefore require treatment or avoidance.

From an engineering perspective, the use of alternative water sources in concrete production is feasible when water quality parameters are maintained within acceptable limits and performance is evaluated across multiple domains. However, reliance on compressive strength alone may lead to misleading conclusions, particularly in cases where durability-related properties are adversely affected. The proposed framework provides a structured approach for field application, enabling engineers to assess water suitability based on both measurable performance indicators and underlying material behavior.

In practical applications, it is recommended that water sources be pre-evaluated using key chemical parameters and validated through performance-based testing when necessary. For moderate impurity conditions, mix design adjustments and controlled replacement strategies may be implemented to mitigate potential adverse effects. For high impurity levels, appropriate treatment or dilution should be applied prior to use to ensure compliance with performance requirements.

## 7. Research Synthesis, Practical Applicability, and Research Trends

Building upon the mechanistic interpretation framework (Section 4.2), the classification of water quality regimes (Section 4.1), and the proposed engineering evaluation framework (Section 5), this section synthesizes the broader implications of the reviewed literature and identifies remaining gaps and future research directions. While this study addresses key limitations by integrating performance, mechanism, and application, several challenges persist in achieving a fully standardized and comprehensive approach to evaluating water quality effects in concrete.

### 7.1. Research Synthesis and Identified Gaps

The reviewed literature consistently demonstrates that the influence of water source quality on concrete performance is governed primarily by chemical composition, impurity concentration, and treatment level, rather than by the simple classification of water as potable or non-potable. Across multiple studies, treated alternative water sources—such as recycled wash water, treated wastewater, and greywater—have been shown to produce comparable compressive strength to conventional potable water when quality parameters remain within acceptable limits.

However, a closer examination of the literature reveals that this apparent agreement is largely limited to strength-based evaluation, with compressive strength dominating as the principal performance indicator. While this provides a useful baseline for feasibility, it also exposes a critical limitation: mechanical strength alone does not fully represent concrete performance, particularly with respect to long-term durability and service life.

In contrast to strength results, findings related to fresh properties and durability performance are considerably more variable and sensitive to water quality variations. Parameters such as total dissolved solids (TDS), suspended solids, chlorides, and organic contaminants have been shown to influence workability, setting time, pore structure, and permeability [39,61,62]. Despite these effects, durability-related properties—including chloride penetration, sulfate resistance, carbonation, and permeability—are less consistently investigated and reported, resulting in an incomplete understanding of long-term performance.

Beyond performance metrics, another major limitation lies in the lack of standardized water quality characterization and reporting. While key parameters such as pH, TDS, TSS, chlorides, and sulfates are commonly measured, their reporting is often inconsistent in terms of units, thresholds, and testing procedures [64,65]. This inconsistency restricts cross-study comparison and weakens the ability to establish universal guidelines.

A further gap emerges in the limited integration of microstructural analysis. Although some studies employ techniques such as SEM and XRD, these are typically used as supplementary tools rather than being systematically linked to mechanical and durability outcomes. As a result, the underlying mechanisms by which water impurities affect hydration processes, pore structure development, and degradation pathways remain insufficiently explained [65,66,67].

In addition to the gaps identified from the scientific literature, several research needs are driven by practical industry requirements, including the development of standardized water quality evaluation protocols, durability-based acceptance criteria, long-term field validation studies, and decision-support frameworks that facilitate the safe implementation of alternative water sources in concrete production.

A key finding emerging from the reviewed literature is that concrete performance is governed more strongly by impurity type, concentration, and treatment level than by the nominal classification of the water source itself. Across multiple studies, treated wastewater, greywater, recycled water, and industrial effluents frequently produced comparable mechanical performance when critical water quality parameters remained within acceptable ranges. Conversely, performance degradation was observed when elevated concentrations of suspended solids, chlorides, sulfates, or organic contaminants interfered with hydration processes and transport properties. This observation suggests that future acceptance criteria should transition from source-based classifications toward parameter-based evaluation frameworks that directly assess water quality characteristics and their influence on hydration and durability mechanisms.

Taken together, the literature reveals a clear pattern: while the feasibility of using alternative water sources has been widely demonstrated, the field remains fragmented, strength-centric, and methodologically inconsistent. This highlights the need for a more integrated and mechanism-based approach to evaluating water quality effects in concrete.

### 7.2. Research Trends and Bibliometric Evidence

The research gaps identified in the preceding section are strongly supported by bibliometric trends observed across the reviewed literature. As illustrated in Figure 3, keyword frequency analysis shows that terms such as “wastewater” and “compressive strength” dominate the research landscape, indicating that current studies are largely centered on water reuse and strength-based performance evaluation. The high occurrence of these keywords confirms that compressive strength remains the primary indicator used to assess the feasibility of alternative water sources in concrete production.

This observation is further reinforced by the thematic distribution of research areas presented in Table 9, where water reuse (44.32%) and mechanical performance (35.23%) collectively account for nearly 80% of the total research focus. In contrast, sustainability (13.64%) and advanced material science (6.81%) remain significantly underrepresented. This imbalance quantitatively demonstrates that the current body of literature is heavily oriented toward feasibility validation and short-term strength assessment, while comparatively less attention is given to long-term durability, environmental impact, and microstructural mechanisms.

**Figure 10:**
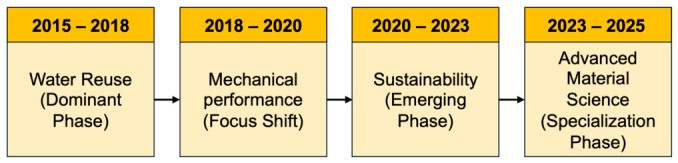
Conceptual illustration of the temporal evolution of research focus in concrete studies u sing alternative water sources.

The temporal evolution of research focus, as illustrated in Figure 10, further highlights this trend. Early studies (2015–2018) were primarily concerned with establishing the feasibility of water reuse in concrete production. This was followed by a period (2018–2020) dominated by mechanical performance validation, where compressive strength became the central evaluation metric. More recent research (2020–2025) shows a gradual shift toward sustainability considerations and advanced material characterization, including durability and microstructural analysis. However, despite this progression, the relatively limited proportion of studies focusing on advanced material science indicates that mechanistic understanding and durability-oriented research remain insufficiently developed.

The bibliometric evidence confirms that the gaps identified in Section 5.1 are not isolated observations but reflect broader trends within the research field. The dominance of strength-based evaluation, combined with the limited emphasis on durability and microstructural behavior, highlights a critical imbalance in current research priorities. This reinforces the need for a more comprehensive and integrated approach to evaluating the effects of water quality on concrete performance.

### 7.3. Recommendations for Future Research

Addressing the identified gaps requires a shift in research approach from isolated, strength-based evaluation toward a more comprehensive and integrated framework. Future studies should move beyond the predominant reliance on compressive strength as the sole performance indicator and instead incorporate a wider range of durability-related properties, including permeability, chloride penetration, carbonation resistance, sulfate attack, shrinkage, and creep. Since these parameters govern long-term structural performance and service life, their inclusion is essential for ensuring the reliability of concrete produced with alternative water sources.

In addition, there is a clear need to strengthen the integration of microstructural and mechanistic analysis within experimental investigations. Techniques such as scanning electron microscopy (SEM), X-ray diffraction (XRD), and pore structure characterization should be more consistently applied and directly linked to observed mechanical and durability outcomes. Establishing these relationships will enable a deeper understanding of how specific water quality parameters influence hydration processes, microstructure development, and long-term degradation mechanisms.

Another critical area for improvement is the standardization of water quality characterization. Future research should adopt consistent methods for measuring and reporting key parameters such as pH, total dissolved solids (TDS), total suspended solids (TSS), chlorides, and sulfates. The use of standardized reporting formats and clearly defined threshold values will enhance comparability across studies and support the development of unified guidelines for the use of alternative water sources in concrete production. Moreover, future studies may also consider conducting a dedicated bibliometric and geographical analysis of research on alternative water sources in concrete production to identify regional research trends, assess the influence of water scarcity and sustainability policies, and highlight geographical areas requiring further investigation.

Furthermore, research efforts should adopt a more holistic evaluation framework that simultaneously considers fresh properties, mechanical performance, durability behavior, and environmental impact. Such an approach would provide a more realistic assessment of material performance under practical conditions and reduce the limitations associated with evaluating isolated properties. This is particularly important given the growing emphasis on sustainable construction practices, where both performance and environmental considerations must be addressed.

Finally, future studies and reviews would benefit from the integration of advanced bibliometric and data-driven analysis techniques. Methods such as keyword co-occurrence mapping and citation network analysis can provide deeper insights into research evolution, identify emerging themes, and systematically highlight underexplored areas. These approaches can complement traditional systematic reviews and contribute to a more structured and evidence-based understanding of research trends. The recommendations are enumerated in Table 10.

## 8. Conclusions

This systematic review demonstrates that the influence of water source quality on concrete performance is primarily governed by the chemical composition, impurity concentration, and treatment level of the mixing water rather than its classification as potable or non-potable. Overall, the findings confirm that properly treated or controlled alternative water sources can be used in concrete production without significant loss of performance, provided that key water quality parameters remain within acceptable limits. Specifically, the following conclusions are drawn in relation to the research objectives:

1. The most critical water quality parameters influencing concrete performance are pH, total dissolved solids (TDS), total suspended solids (TSS), chlorides, and sulfates, as these directly affect cement hydration, setting behavior, and long-term durability. Across the reviewed studies, acceptable performance is generally observed when pH remains within approximately 6.5–8.5, while TDS values below 2000 mg/L are commonly associated with stable hydration behavior. Higher impurity levels, particularly chloride concentrations exceeding ~600 mg/L and sulfate concentrations above 1000 mg/L, are frequently linked to increased porosity, delayed or accelerated hydration, and potential durability risks.
2. Compressive strength remains the most widely used indicator for evaluating the suitability of alternative water sources, with 27 out of 38 reviewed studies employing compressive strength testing as the primary performance metric. Most studies report comparable or improved strength when water quality is properly controlled, with variations typically ranging from −10% to +15% relative to control mixtures. In some cases, strength increases of up to 79% were reported under optimized conditions, while untreated or highly contaminated water sources resulted in reductions of up to 5–33%.
3. Fresh properties and durability-related performance are more sensitive to variations in water quality than compressive strength. Reported results indicate slump reductions ranging from 20% to 30%, particularly in mixtures containing higher concentrations of suspended solids or dissolved salts. Setting time variations range from ±20–25 min under typical conditions to extreme delays exceeding 10 h in cases involving highly contaminated or recycled water. Durability-related indicators, including permeability and resistance to chloride penetration, show greater variability, with some studies reporting acceptable strength despite reductions in long-term durability performance due to increased pore connectivity and transport properties.
4. Variations in experimental methods, including differences in water characterization, mixture design, curing conditions, and testing procedures, contribute significantly to inconsistencies in reported results and limit direct comparison across studies. For example, compressive strength differences of up to ~15–20% are often attributed not only to water quality but also to variations in curing regimes and mixture proportions. This highlights the importance of standardized testing protocols and consistent reporting of water quality parameters.
5. Despite the growing body of research, the current literature remains predominantly strength-oriented, with only 14 out of 38 studies evaluating durability and fewer than 10 studies incorporating microstructural analysis. This study contributes a mechanism-based framework linking water chemistry to hydration, microstructural development, and durability performance, providing a more comprehensive basis for evaluating alternative water sources beyond compressive strength alone. However, limitations arise from the lack of standardized water quality reporting, variability in experimental conditions, and inconsistent inclusion of durability-related assessments, which restrict direct comparison across studies, while the exclusion of seawater and corrosion-dominated environments limits applicability to non-marine conditions. Future research should focus on standardizing water quality thresholds, expanding long-term durability investigations, and integrating microstructural characterization with mechanical and transport properties to support performance-based evaluation of alternative water sources.
6. Future research should therefore address not only the scientific uncertainties identified in the literature but also the practical requirements of industry stakeholders, particularly with respect to standardization, durability assurance, quality control procedures, and the large-scale implementation of alternative water sources in concrete production.

## Figures and Tables

**Table 1:** Literature Search Strategy and Initial Retrieval Results.

Database	Filters Applied	Results Retrieved	Full Search String
Web of Science		50	(“mixing water” OR “water quality” OR “water source quality”) AND concrete AND (durability OR “mechanical properties” OR strength OR workability)
IEEE	2016–2026 Engineering Material science Has PDF	15	(“water quality” OR “mixing water”) AND (concrete) AND (cement) AND (strength) AND (durability) AND (workability) AND (wastewater)
Scopus	2016–2026 Engineering Material science	29	(“water quality” AND concrete AND cement AND “mixing water” AND strength AND durability AND workability AND wastewater)
Science Direct	2016–2026 Engineering Material science	43	“water quality” AND concrete AND cement AND “mixing water” AND strength AND durability AND workability AND wastewater
PubMed	2016–2026	38	(“water source quality” OR “water quality” OR “wastewater”) AND (concrete OR cement) AND (durability OR workability)

**Table 2:** Criteria for Relevance Assessment and Study Inclusion.

Score	Level of Relevance	Criteria Description
5	Very High	Provides detailed analysis of measurable water quality parameters and their effects on concrete performance.
4	High	Examines water quality effects on concrete performance with limited analysis.
3	Moderate	Discusses water use in concrete, but water quality is not the main focus.
2	Low	Mentions water quality without clear relationship to concrete performance.
1	Very Low	Provides background information without analyzing the impact of water quality on concrete performance.

**Table 4:** Summary of the Effects of Different Water Sources on Concrete Properties.

Water Source Category	Reference	Mixing Water	Slump/ Workability	Setting Time	Compressive Strength	Tensile Strength	Flexural Strength	% WW in Mixing Water
Potable/Control Water Source	[50]	Drinking water, Groundwater, Sewage water	117–139 mm slump	–	Sewage water −25–33% vs. potable water	–	–	–
	[51]	Domestic water, Recycled water	45–52 mm slump	Initial set 11 h 54 min–20 h 45 min	21.8–24.1 MPa at 28 days	–	–	–
	[52]	Potable, River, Rainwater	10–25 mm slump	Initial 168–193 min	River water 97.6%, deep well water 91.1%, rainwater 83.7% of control	Slight reduction	Slight reduction	–
Treated Wastewater and Effluents	[55]	Potable water and STP-treated effluent (E1, E2)	–	Initial setting 30–35 min, final setting 180–200 min (within IS limits)	Maximum 34.79 MPa (28 days) when potable water used for casting	–	–	Effluent used for casting and/or curing combinations (0–100%)
	[44]	Tap water and tertiary treated wastewater (Jebel Ali STP)	Slump decreased from 65 mm (tap water) to 46 mm (tertiary treated wastewater) (~29% decrease)	–	18.5 MPa (tap water) vs. 24.62 MPa (tap mix + WW curing); 14 days: up to 37.3 MPa; 21 days: up to 40.5 MPa (≈33–70% increase depending on water use)	2.83 MPa (tap) → 2.93 MPa (WW mix) (~4.2% increase at 28 days)	4.2 MPa (tap) → 3.8 MPa (WW mix) (~8% decrease at 28 days)	0–100% wastewater (mixing and/or curing)
	[56]	Raw wastewater, Treated wastewater	Slump flow similar to control	–	Early strength −32–35%, ≈control at 90 days	≤10% reduction	≤18–20% reduction	–
	[49]	Tap water, Treated wastewater	–	–	−15% to +8% vs. control depending on treatment	–	–	–
	[62]	Tap water, Treated wastewater	Lower slump with treated wastewater	Initial set ~3.5 min later	62–71 MPa (28 d), 66–77 MPa (150 d)	≥3 MPa (reduced under long exposure)	–	100%
	[63]	Bore water, Treated domestic wastewater, Polished filtered wastewater	Slump ≈ 4–5 in (100–125 mm) for all mixes	Not significantly reported	~15% lower compressive strength at 28 days	20–25% higher tensile strength for treated and polished wastewater compared with bore water	Flexural strength ≈ similar to bore water (difference < 2 MPa)	100% replacement tested
	[63]	Tap water, Treated wastewater	80 mm (tap), 60 mm (tertiary WW), 40 mm (secondary WW)	–	85–94% of control strength	–	−7.7–13.1%	100%
	[64]	Drinking water, Treated wastewater	150–190 mm slump	Slight delay	Early strength ~83% control; later ≈ control	−5–37%	–	50–100%
	[65]	Raw sewage, Treated effluent	Slight slump reduction	Initial set 128 → 110 min	7-day strength +40%; later ≈ control	–	–	–
	[54]	Wastewater effluent	–	Accelerated hydration	−21–37% strength	–	–	–
	[53]	Potable water, Treated wastewater	Slump reduced 13–20%	–	~10% reduction	~19% reduction	–	–
Greywater	[57]	Raw grey water (RGW) and Treated grey water (TGW)	Slump decreased by 3–3.5 cm	Initial setting time increased by +20 min (RGW) and +25 min (TGW) compared with distilled water	28–120 days: TGW ≈ no change; RGW reduced strength by 7.7–13.9%	–	–	50–100% grey water replacement
	[48]	Tap water, Greywater	Slight slump reduction	Initial set delayed ~20–25 min	Greywater +11.8%; combined water +3.3%	–	–	–
Industrial Wastewater	[47]	Tap water, Industrial wastewater	–	Slight delay	~2% reduction vs. tap water	–	–	–
	[58]	Produced water	Flow reduced ~20%	Initial set −25%	20.4 MPa (0% WW), 34.5 MPa (80% WW), 23.7 MPa (100% WW)	–	–	20–100%
	[59]	Distillery wastewater	Acceptable workability	Minor variation	Comparable or slightly improved strength	Cylinder load 230–330 kN	Beam load 3320–3940 kgf	20%
	[66]	Coal mining wastewater	Good workability	–	+15.6% (50% WW) and +79% (100% WW)	1.58–2.12 MPa	+8.7–17.4% flexural capacity	50–100%
	[43]	Tap water + Concrete wash water from ready-mix trucks and batching plant	Slump 12–15 cm for all mixes	Setting time 140–150 min; longer when 50–70% wash water used	~40.6 MPa (100% wash water) vs. ~45 MPa (100% tap water) at 90 days	Differences ≤ 1 MPa, not significant	Differences ≤ 0.58 MPa, not significant	0–100% wash water replacement
Natural Water Sources	[46]	Distilled, Tap, River, Wastewater, Lake water	15.5–17 cm slump	–	River water +15–29%; wastewater +14–50% early then −2.3%; lake water −6–22% later ages	–	–	100%
	[60]	River water	Slight slump reduction	–	16.04 MPa vs. 18.22 MPa control	–	–	–
	[52]	Potable, River, Rainwater	10–25 mm slump	Initial 168–193 min	River water 97.6%, deep well water 91.1%, rainwater 83.7% of control	Slight reduction	Slight reduction	–
Concrete Plant/Wash Water	[45]	Waste wash-water (WWW) from ready-mix concrete plant (raw, settling pond, filtered, filtered-neutralized)	Raw WWW: very low slump; Filtered WWW: slight slump reduction	-	28–90 days: no significant difference from control for filtered or settling-pond water; Raw WWW: reduction up to −12.7%	-	-	Up to 75–100% recycled wash-water
	[61]	Concrete wash wastewater	Slump increased (48 mm vs. 30 mm)	Accelerated	Similar strength (≤3% difference)	–	6.7–10.2 MPa	100%
	[67]	Concrete plant wastewater	Slump decreased with higher WW	–	Peak strength at 75% WW (~5.4% higher than control)	–	Reduced flexural loss	0–75%

**Table 5:** Classification of Water Quality Regimes and Associated Concrete Performance.

Impurity Level	Hydration Behavior	Microstructure	Transport Properties	Durability Performance
Low Impurity	Controlled hydration	Dense, well-formed C–S–H structure	Low permeability and diffusivity	High durability; strong resistance to degradation
Moderate Impurity	Variable hydration (acceleration or retardation depending on composition)	Mixed pore structure; partial refinement or disruption	Moderate transport properties	Variable durability; dependent on mixture design and impurity type
High Impurity	Disrupted or delayed hydration; formation of undesirable products	Porous structure; weak bonding and high porosity	High permeability and ion transport	Poor durability; increased susceptibility to chloride ingress, sulfate attack, and degradation

**Table 6:** Comparative Relationship Between Water Quality and Concrete Performance.

Water Source Condition	Typical Water Characteristics	Workability	Compressive Strength	Durability Performance	Dominant Mechanism
Treated wastewater	Controlled TDS, reduced organics, regulated pH	Slight reduction or stable	Comparable or improved	Generally acceptable	Improved hydration and refined pore structure
Treated greywater	Reduced suspended solids, moderate impurities	Slight reduction	Comparable	Limited data available	Reduced interference with hydration reactions
Treated concrete wash water	Fine particles, residual cement content	Stable or slight reduction	Comparable	Acceptable under controlled conditions	Filler effect and particle packing
Recycled water (controlled)	Moderate dissolved solids, filtered contaminants	Slight reduction	Comparable to control	Moderate performance	Balanced hydration and microstructural stability
Untreated wastewater	High TDS, organics, suspended solids	Significant reduction	Reduced	Poor durability	Increased porosity and delayed hydration
Industrial wastewater	Variable contaminants (chemicals, oils)	Highly variable	Variable or reduced	Potential risks	Chemical interference
Sewage water (untreated)	High organic content	Variable	Significant reduction	Poor durability	Weak microstructure
Natural non-potable water	Moderate impurities	Slight variation	Comparable or slightly reduced	Generally acceptable	Minor influence on hydration

**Table 7:** Multi-Dimensional Evaluation Framework for Water Quality in Concrete.

Evaluation Domain	Key Parameters	Purpose	Engineering Significance
Fresh Properties	Slump, setting time	Assess workability and early-stage behavior	Ensures constructability and proper placement
Mechanical Performance	Compressive, tensile, flexural strength	Evaluate structural capacity	Confirms load-bearing performance
Durability Indicators	Permeability, water absorption, chloride penetration	Assess long-term performance	Determines service life and resistance to degradation
Microstructural Characteristics	Pore structure, C–S–H formation, porosity	Explain internal behavior	Provides mechanistic validation of observed performance

**Table 8:** Classification of Impurity Levels in Alternative Water Sources for Concrete Production.

Impurity Level	TDS (mg/L)	Chloride (mg/L)	Sulfate (mg/L)	pH Range	TSS (mg/L)	Expected Performance
Low Impurity	<500	<200	<200	6.5–8.5	<50	Comparable to potable water; stable hydration; high durability
Moderate Impurity	500–2000	200–600	200–1000	6.0–9.0	50–500	Variable performance; possible strength retention but durability risk
High Impurity	>2000	>600	>1000	<6 or >9	>500	Reduced strength and durability; disrupted hydration; high permeability

**Table 9:** Relative percentage distribution of major research themes identified through bibliometric keyword analysis of the reviewed literature.

Category	Relative %
Water reuse	44.32
Mechanical performance	35.23
Sustainability	13.64
Advanced material science	6.81

**Table 10:** Summary of Identified Research Gaps and Recommended Future Directions.

Identified Research Gap	Recommended Direction
Overreliance on compressive strength as the primary performance indicator	Expand evaluation to include durability-related properties such as permeability, chloride penetration, carbonation, sulfate resistance, shrinkage, and creep
Limited investigation of durability and long-term performance	Conduct long-term studies focusing on service life behavior and environmental exposure conditions
Insufficient integration of microstructural analysis	Incorporate SEM, XRD, and pore structure analysis and link findings to performance outcomes
Inconsistent reporting of water quality parameters	Standardize measurement and reporting of key parameters (pH, TDS, TSS, chlorides, sulfates)
Fragmented evaluation of concrete properties	Develop holistic frameworks that assess fresh, mechanical, durability, and environmental performance simultaneously
Limited focus on sustainability and environmental impact	Integrate life-cycle assessment (LCA) and sustainability metrics into experimental studies
Underutilization of advanced bibliometric methods	Apply keyword mapping and citation network analysis to better understand research evolution and gaps

## Data Availability

No new data were created or analyzed in this study. Data sharing is not applicable to this article.

## Supplementary Materials

The following supporting information can be downloaded at: https://www.mdpi.com/article/10.3390/ma19143077/s1: PRISMA 2020 checklist.

## Nomenclature

**Symbol/Abbreviation**	**Description**
C	CaO (Calcium oxide)
S	SiO_2_ (Silicon dioxide)
A	Al_2_O_3_ (Aluminum oxide)
F	Fe_2_O_3_ (Iron oxide)
H	H_2_O (Water)
Ŝ	SO_3_ (Sulfate)
C_3_S	Tricalcium silicate (3CaO·SiO_2_)
C_2_S	Dicalcium silicate (2CaO·SiO_2_)
C_3_A	Tricalcium aluminate (3CaO·Al_2_O_3_)
C_4_AF	Tetracalcium aluminoferrite (4CaO·Al_2_O_3_·Fe_2_O_3_)
C–S–H	Calcium silicate hydrate
CH	Calcium hydroxide
AFt	Ettringite phase
AFm	Monosulfate phase
TDS	Total dissolved solids
TSS	Total suspended solids
COD	Chemical oxygen demand
BOD	Biological oxygen demand
SEM	Scanning electron microscopy
XRD	X-ray diffraction
TGA	Thermogravimetric analysis
ASTM	American Society for Testing and Materials
WW	Wastewater
NTU	Nephelometric Turbidity Unit

## References

[1] Robles K.P.V., Yee J.J., Kee S.H. (2025). Simulation-Based Assessment of the Impact of Internal and Surface-Breaking Cracks on Reinforced Concrete Electrical Resistivity. Proceedings of the 7th International Conference on Civil Engineering and Architecture.

[2] Li M.L.L., Kee S.-H., Monjardin C.E.F., Robles K.P.V. (2025). Numerical and Experimental Correlation Between Half-Cell Potential and Steel Mass Loss in Corroded Reinforced Concrete. Materials.

[3] Hover K.C. (2011). The Influence of Water on the Performance of Concrete. Constr. Build. Mater..

[4] Aïtcin P.C. (2016). Water and Its Role on Concrete Performance. Science and Technology of Concrete Admixtures.

[5] Al-Jabari M. (2022). Introduction to Concrete Chemistry. Integral Waterproofing of Concrete Structures: Advanced Protection Technologies of Concrete by Pore Blocking and Lining.

[6] Marchon D., Flatt R.J. (2016). Mechanisms of Cement Hydration. Science and Technology of Concrete Admixtures.

[7] Chandio M.A., Shaikh S., Chandio W.M. (2024). The Effect of Different Water Sources on the Quality of Concrete for Infrastructure Projects. Kashf J. Multidiscip. Res..

[8] Wu H.C. (2014). Re-Examination of Cement Hydration: Sol-Gel Process. Adv. Cem. Res..

[9] Bentz D.P. (2007). Cement Hydration: Building Bridges and Dams at the Microstructure Level. Mater. Struct. Mater. Constr..

[10] Suraneni P., Flatt R.J. (2015). Micro-Reactors to Study Alite Hydration. J. Am. Ceram. Soc..

[11] Bouaich F.Z., Maherzi W., El-hajjaji F., Abriak N.E., Benzerzour M., Taleb M., Rais Z. (2022). Reuse of Treated Wastewater and Non-Potable Groundwater in the Manufacture of Concrete: Major Challenge of Environmental Preservation. Environ. Sci. Pollut. Res..

[12] Robles K.P.V., Monjardin C.E.F. (2025). Assessment and Monitoring of Groundwater Contaminants in Heavily Urbanized Areas: A Review of Methods and Applications for Philippines. Water.

[13] Ahmed A., Ahmed O., Al-Fakih A., Muhit I.B. (2025). A Comprehensive Review on the Use of Reclaimed Wastewater in Cementitious Materials: Fresh, Mechanical, Microstructure, and Durability Aspects. Arab. J. Sci. Eng..

[14] Wang J., Xie J., Wang Y., Liu Y., Ding Y. (2020). Rheological Properties, Compressive Strength, Hydration Products and Microstructure of Seawater-Mixed Cement Pastes. Cem. Concr. Compos..

[15] Farid H., Mansoor M.S., Shah S.A.R., Khan N.M., Shabbir R.M.F., Adnan M., Arshad H., Haq I.U., Waseem M. (2019). Impact Analysis of Water Quality on the Development of Construction Materials. Processes.

[16] Awoyera P.O., Awobayikun O., Gobinath R., Ugwu E.I. (2020). Rheological, Mineralogical and Strength Variability of Concrete Due to Construction Water Impurities. Int. J. Eng. Res. Afr..

[17] Kaboosi K., Fadavi M., Setayesh E. (2019). The Feasibility Study of the Use of Briny Groundwater and Zeolite in the Plain Concrete Mix Design in the Different Cement Contents. Civ. Environ. Eng..

[18] Wang X., Yu H., Tan Y., Wu C., Wu P., Ma H., Ding Z., Liu L. (2025). Mechanical Properties, Corrosion Damage Evolution Laws, and Durability Deterioration Indicators of High-Performance Concrete Exposed to Saline Soil Environment for 8 Years. Materials.

[19] Li B., Mao J., Lv J., Zhou L. (2016). Effects of Micropore Structure on Hydration Degree and Mechanical Properties of Concrete in Later Curing Age. Eur. J. Environ. Civ. Eng..

[20] Blouch N., Kazmi S.N.H., Metwaly M., Akram N., Mi J., Hanif M.F. (2025). Towards Sustainable Construction: Experimental and Machine Learning-Based Analysis of Wastewater-Integrated Concrete Pavers. Sustainability.

[21] Çomak B. (2018). Effects of Use of Alkaline Mixing Waters on Engineering Properties of Cement Mortars. Eur. J. Environ. Civ. Eng..

[22] Ghasemi S., Nikudel M.R., Zalooli A., Khamehchiyan M., Alizadeh A., Yousefvand F., Ghasemi A.M.R. (2022). Durability Assessment of Sulfur Concrete and Portland Concrete in Laboratory Conditions and Marine Environments. J. Mater. Civ. Eng..

[23] Zhang H. (2025). Advances and Challenges in Concrete Repair Materials: A Review. Adv. Res..

[24] Agwe T.M., Tibenderana P., Twesigye-Omwe M.N., Mbujje J.W., Abdulkadir S.T. (2022). Concrete Production and Curing with Recycled Wastewater: A Review on the Current State of Knowledge and Practice. Adv. Civ. Eng..

[25] Azeem A., Ahmad S., Hanif A. (2023). Wastewater Utilization for Concrete Production: Prospects, Challenges, and Opportunities. J. Build. Eng..

[26] Maddikeari M., Das B.B., Tangadagi R.B., Roy S., Nagaraj P.B., Ramachandra M.L. (2024). A Comprehensive Review on the Use of Wastewater in the Manufacturing of Concrete: Fostering Sustainability through Recycling. Recycling.

[27] Nikookar M., Brake N.A., Adesina M., Rahman A., Selvaratnam T. (2023). Past, Current, and Future Re-Use of Recycled Non-Potable Water Sources in Concrete Applications to Reduce Freshwater Consumption—A Review. Clean. Mater..

[28] Gokulanathan V., Arun K., Priyadharshini P. (2021). Fresh and Hardened Properties of Five Non-Potable Water Mixed and Cured Concrete: A Comprehensive Review. Constr. Build. Mater..

[29] Harishbabu J., Saboo N., Kar S.S. (2024). Use of Non-Potable Water Sources in Pavement Construction: A Review. Constr. Build. Mater..

[30] Reddy Babu G., Madhusudana Reddy B., Venkata Ramana N. (2018). Quality of Mixing Water in Cement Concrete “a Review”. Mater. Today Proc..

[31] Al-Jabri K.S., AL-Saidy A.H., Taha R., AL-Kemyani A.J. (2011). Effect of Using Wastewater on the Properties of High Strength Concrete. Procedia Eng..

[32] Asadollahfardi G., Asadi M., Jafari H., Moradi A., Asadollahfardi R. (2015). Experimental and Statistical Studies of Using Wash Water from Ready-Mix Concrete Trucks and a Batching Plant in the Production of Fresh Concrete. Constr. Build. Mater..

[33] Elsayed K.M.N.I., Guico G.B.F., Rustum R. (2023). Concrete behavior using recycled wastewater. Int. J. GEOMATE.

[34] Ghrair A.M., Heath A., Paine K., Al Kronz M. (2020). Waste Wash-Water Recycling in Ready Mix Concrete Plants. Environments.

[35] Humood S.H., Jassim F.N., Sulaiman E.A., Al-Gasham T.S. (2025). Effect of Different Types of Water on Workability and Compressive Strength of Concrete. Math. Model. Eng. Probl..

[36] Kaboosi K., Emami K. (2019). Interaction of Treated Industrial Wastewater and Zeolite on Compressive Strength of Plain Concrete in Different Cement Contents and Curing Ages. Case Stud. Constr. Mater..

[37] Kaboosi K., Kaboosi F., Fadavi M. (2020). Investigation of Greywater and Zeolite Usage in Different Cement Contents on Concrete Compressive Strength and Their Interactions. Ain Shams Eng. J..

[38] Kim J.S., Oh S.E., Kim S.Y., Pyeon G., Maeng S., Chung S.Y. (2025). Effects of Treated Wastewater from Recycled Aggregate Processing on the Microstructure and Mechanical Properties of Cement Mortar. Constr. Build. Mater..

[39] Kokoszka W. (2019). Impact of Water Quality on Concrete Mix and Hardened Concrete Parameters. Civ. Environ. Eng. Rep..

[40] Lu Q., Fan Z., Zhou X., Peng Z., Gao Z.F., Deng S., Han W., Jin Z., Chen X. (2023). Water-Saving Optimization Design of Aggregate Processing Plant and Recycled Water Utilization for Producing Concrete. Constr. Build. Mater..

[41] Mohe N.S., Shewalul Y.W., Agon E.C. (2022). Experimental Investigation on Mechanical Properties of Concrete Using Different Sources of Water for Mixing and Curing Concrete. Case Stud. Constr. Mater..

[42] Nasseralshariati E., Mohammadzadeh D., Karballaeezadeh N., Mosavi A., Reuter U., Saatcioglu M. (2021). The Effect of Incorporating Industrials Wastewater on Durability and Long-Term Strength of Concrete. Materials.

[43] Soltanianfard M.A., Hojat Jalali H., Shah S.P. (2025). Concrete Produced with Wastewater from Early-Stages of Treatment: Performance and Enhancement through Supplementary Cementitious Materials. Constr. Build. Mater..

[44] Chaudhari R.S. (2024). Unlocking Concrete Strength: Empowering Sustainable Practices through Effluent Recycling in Casting and Curing. Mater. Today Proc..

[45] Jahandideh E., Asadollahfardi G., Akbardoost J., Salehi A. (2024). The Effect of Chemical Oxygen Demand of Domestic Wastewater on Workability, Mechanical, and Durability of Self- Compacting Concrete. Case Stud. Constr. Mater..

[46] Ghrair A.M., Al-Mashaqbeh O.A., Sarireh M.K., Al-Kouz N., Farfoura M., Megdal S.B. (2018). Influence of Grey Water on Physical and Mechanical Properties of Mortar and Concrete Mixes. Ain Shams Eng. J..

[47] Nikookar M., Brake N.A., Asli H.H., Adesina M., Rahman A., Selvaratnam T., Bradley R.K. (2023). Durability, Workability, and Setting Time of Cementitious Systems Containing Chloride-Rich Oil and Gas Production Wastewater. Constr. Build. Mater..

[48] Pabale A.R., Jadhav M.V. (2025). AI Based Investigation on Concrete Using Distillery Waste as Partial Replacement to Potable Water. Proceedings of the International Conference on Engineering, Technology and Management, ICETM 2025.

[49] Morfe R. (2025). Comparative study of concrete compressive strength using potable and river water with multiple linear regression prediction. Int. J. GEOMATE.

[50] Tsardaka E.C., Anastasiou E.K., Karanafti A., Ferriz-Papi J.A., Valentin J., Theodosiou T. (2026). Influence of Cement Type on the Performance and Durability of Cement Paste and Concrete with Wastewater. Materials.

[51] Ahmed S., Alhoubi Y., Elmesalami N., Yehia S., Abed F. (2021). Effect of Recycled Aggregates and Treated Wastewater on Concrete Subjected to Different Exposure Conditions. Constr. Build. Mater..

[52] Arooj M.F., Haseeb F., Butt A.I., Irfan-Ul-Hassan D.M., Batool H., Kibria S., Javed Z., Nawaz H., Asif S. (2021). A Sustainable Approach to Reuse of Treated Domestic Wastewater in Construction Incorporating Admixtures. J. Build. Eng..

[53] Morgado J., Rosales J., de Brito J., Mendes M.P., Machini B., Bravo M. (2024). Performance of Concrete with Treated Wastewater and Recycled Aggregates. J. Build. Eng..

[54] Naik P.A., Udayakumar G., Rao C.V., Marathe S. (2020). Influence of STP Treated and Reed Bed Treated Domestic Wastewater on Properties of Mortar and Concrete Mixes. Int. J. Eng. Trends Technol..

[55] Kısa K.O., Ülger T., Cavuslu M. (2025). Utilization of Coal Mining Wastewater in Concrete Production: Experimental and Finite Element Simulation of Flexural Performance in Reinforced Concrete Beams. Structures.

[56] Meena K., Luhar S. (2019). Effect of Wastewater on Properties of Concrete. J. Build. Eng..

[57] Yao X., Xi J., Guan J., Liu L., Shangguan L., Xu Z. (2022). A Review of Research on Mechanical Properties and Durability of Concrete Mixed with Wastewater from Ready-Mixed Concrete Plant. Materials.

[58] Civil Engicon Team Concrete Workability: Key Factors, Effects & How to Improve It for Stronger Structures. https://www.civilengicon.com/2023/10/workability-of-concrete-types-factors.html.

[59] Sheikh Hassani M., Matos J.C., Zhang Y.X., Teixeira E. (2023). Concrete Production with Domestic and Industrial Wastewaters—A Literature Review. Struct. Concr..

[60] Yao X., Xu Z., Guan J., Liu L., Shangguan L., Xi J. (2022). Influence of Wastewater Content on Mechanical Properties, Microstructure, and Durability of Concrete. Buildings.

[61] Aldayel Aldossary M.H., Ahmad S., Bahraq A.A. (2020). Effect of Total Dissolved Solids-Contaminated Water on the Properties of Concrete. J. Build. Eng..

[62] Dey G., Ganguli A., Bhattacharjee B. Estimation of Degree of Moisture Saturation in Cement Concrete Using Electrical Response at Low Radio Frequencies. Proceedings of the NDE 2019—Conference & Exhibition.

[63] Jan F., Min-Allah N., Düştegör D. (2021). Iot Based Smart Water Quality Monitoring: Recent Techniques, Trends and Challenges for Domestic Applications. Water.

[64] Vandegrift J., Hooper J., da Silva A., Bell K., Snyder S., Rock C.M. (2019). Overview of Monitoring Techniques for Evaluating Water Quality at Potable Reuse Treatment Facilities. J. Am. Water Works Assoc..

[65] Tong P., Wang Y., Wang M., Zhang X., Su H., Li Z., Zhou S., Zhao C., Zhang X., Zhang J. (2026). Synergistic Effects of Sulfate, Magnesium, and Bicarbonate Ions in Mine Water on the Hydration, Microstructure, and Long-Term Durability of Portland Cement Paste. Miner. Eng..

[66] Sun M., Yu R., Jiang C., Fan D., Shui Z. (2022). Quantitative Effect of Seawater on the Hydration Kinetics and Microstructure Development of Ultra High Performance Concrete (UHPC). Constr. Build. Mater..

[67] Galan I., Perron L., Glasser F.P. (2015). Impact of Chloride-Rich Environments on Cement Paste Mineralogy. Cem. Concr. Res..

