# Supplementary material for: Influence of Water Source Quality on Concrete Performance: A Mechanism-Based Systematic Review and Engineering Evaluation Framework
Source: Materials (Basel). 2026 Jul 17;19(14):3077. doi: 10.3390/ma19143077 (PMC13413510; doi:10.3390/ma19143077)
Supplement: Supplementary file 1 [file materials-19-03077-s001.zip › materials-4320761-supplementary.pdf]

PRISMA 2020 Checklist

Section	Item	PRISMA 2020 Checklist Item	Location in Manuscript
TITLE	1	Identify the report as a systematic review.	Title
ABSTRACT	2	See PRISMA 2020 for Abstracts checklist.	Abstract
INTRODUCTION	3	Describe rationale for the review.	Introduction
INTRODUCTION	4	State objectives or questions addressed by the review.	End of Introduction
METHODS	5	Specify inclusion and exclusion criteria and grouping.	Section 2.3
METHODS	6	Specify databases and search dates.	Section 2.2, Table 1
METHODS	7	Present full search strategies.	Section 2.2, Table 1
METHODS	8	Describe study selection methods.	Section 2.3
METHODS	9	Describe data collection methods.	Section 2.3
METHODS	10	List outcomes and variables collected.	Section 2.4
METHODS	11	Describe risk of bias assessment methods.	Not performed; relevance scoring used
METHODS	12	Specify effect measures.	Not applicable
METHODS	13	Describe synthesis methods.	Section 2.4
METHODS	14	Describe reporting bias assessment.	Not performed
METHODS	15	Describe certainty assessment.	Not performed
RESULTS	16	Describe search and selection process.	Figure 2
RESULTS	17	Present characteristics of included studies.	Tables 3–4
RESULTS	18	Present risk of bias assessments.	Not performed
RESULTS	19	Present results of individual studies.	Table 4
DISCUSSION	23	Interpret results and discuss limitations.	Discussion
OTHER INFORMATION	24	Provide registration and protocol information.	Not registered
OTHER INFORMATION	25	Describe sources of support.	Funding statement
OTHER INFORMATION	26	Declare competing interests.	Conflict of Interest section
OTHER INFORMATION	27	Report availability of data and materials.	Data Availability Statement
